# Nanotechnology-based immunotherapy: integrating Artificial Intelligence (AI) with current strategies in combating brain cancer disease

**DOI:** 10.1186/s43046-026-00394-3

**Published:** 2026-08-18

**Authors:** Alia Syazana Mohd Roslei, Hasniza Zaman Huri, Dyah Ayu Oktavianie A. Pratama, Ahmad Khusairi Azemi, Zulhisyam Abdul Kari, Md Yasin Ina-Salwany, Najihah Mohd Hashim, Mohd Faizal Ghazali, Abdin Shakirin Mohamad Norpi, Muhammad Fauzi Abd Jalil, Asif Nawaz, Said AlGhenaimi, Mazlan Mohamed, Muhammad Luqman Nordin

**Affiliations:** 1 Department of Pharmaceutical Technology, Faculty of Pharmacy, University of Malaya, Kuala Lumpur, 50603 Malaysia; 2 Department of Clinical Pharmacy and Pharmacy Practice, Faculty of Pharmacy, University Malaya, Kuala Lumpur, 50603 Malaysia; 3 Department of Pathology, Faculty of Veterinary Medicine, University of Brawijaya, Malang, East Java 65151 Indonesia; 4 Higher Institution Centre of Excellence (HICoE), Institute of Climate Adaptation and Marine Biotechnology, Universiti Malaysia Terengganu, Kuala Terengganu, Terengganu 21030 Malaysia; 5 Department of Agricultural Sciences, Faculty of Agro-Based Industry, Universiti Malaysia Kelantan, Jeli Campus, Jeli, Kelantan 17600 Malaysia; 6 Laboratory of Aquatic Animal Health and Therapeutic (AquaHealth), Institute of Bioscience, Universiti Putra Malaysia, Seri Kembangan, Selangor 43400 Malaysia; 7 Department of Aquaculture, Faculty of Agriculture, Universiti Putra Malaysia, UPM, Serdang, Selangor 43400, Malaysia; 8 Department of Pharmaceutical Chemistry, Faculty of Pharmacy, Universiti Malaya, Kuala Lumpur , 50603 Malaysia; 9 Centre for Natural Products Research and Drug Discovery (CENAR), Universiti Malaya, Kuala Lumpur, 50603 Malaysia; 10 Faculty of Veterinary Medicine, Universiti Sultan Zainal Abidin, Kuala Terengganu, Besut, Terengganu 22200 Malaysia; 11 Faculty of Pharmacy and Health Sciences, Universiti Kuala Lumpur, Royal College of Medicine Perak, Ipoh, Perak 30450 Malaysia; 12 Department of Medical Laboratory Sciences, College of Applied and Health Sciences, A’Sharqiyah University, Ibrā’, 400 Oman; 13 Faculty of Artificial Intelligence and Cyber Security (FAIX), Technical University of Malaysia Malacca, Hang Tuah Jaya, Malacca, 76100 Malaysia; 14 Centre of Excellence for Food Security and Intelligent Farming, University of Malaya, Kuala Lumpur, 50603 Malaysia; 15 Department of Pharmacology, Faculty of Medicine, Universiti Malaya Research Centre for Pharmaceuticals and Advanced Therapeutics (UBAT),Universiti Malaya, Kuala Lumpur, 50603 Malaysia

**Keywords:** Glioblastoma, Nanotechnology, Immunotherapy, Cancer vaccine, Artificial Intelligence

## Abstract

Brain cancer is one of the most challenging malignancies and a major contributor to worldwide morbidity and mortality. Glioblastoma, the most aggressive adult brain tumor, is associated with poor prognosis despite conventional therapies such as surgery, chemotherapy, and radiotherapy, which often result in severe toxicity and long-term side effects. Immunotherapy holds the potential to provide durable and specific anti-tumor responses; however, its success in brain cancer is hindered by obstacles such as the blood-brain barrier, immunosuppressive tumor microenvironment, and tumor heterogeneity. Nanomedicine offers a powerful approach to overcoming these barriers through targeted and efficient drug delivery. Nanotechnology-based platforms, including lipid-based, polymeric, and inorganic nanoparticles, have demonstrated superior therapeutic efficacy compared to free drugs, with several formulations advancing into clinical trials. Among these, nanotechnology-enabled vaccines represent an emerging frontier, capable of enhancing antigen presentation, stimulating strong immune responses, and overcoming tumor-induced immunosuppression. By combining the precision of nanocarriers with the long-lasting protection of vaccines, nano-vaccines hold great potential to transform brain cancer immunotherapy. Recent studies also suggest that integrating artificial intelligence (AI) with nanotechnology could further enhance the design, targeting, and effectiveness of immunotherapies. Moreover, AI is revolutionizing treatment development by enabling the prediction of immunogenicity, immune responses, and optimizing formulation design and dosing strategies. These advancements collectively accelerate the development and enhance the precision and efficiency of immunotherapy. Hence, this review discusses current nanotechnology-based immunotherapies and highlights the emerging role of integrating AI in vaccine development as next-generation strategies for improving outcomes in brain cancer treatment.

**Supplementary Information:**

The online version contains supplementary material available at 10.1186/s43046-026-00394-3.

## Introduction

Brain cancers are relatively rare and account for only 1 to 2% of cancer incidence. However, they are linked to significant morbidity and mortality due to their deadly malignancy, with a poor survival rate [1]. Based on the 2020 Global Cancer Observatory (GLOBOCAN), in 2020, brain and CNS cancers were estimated to cause 321,731 new cases and 248,500 deaths worldwide. In the last three decades, there has been an increasing trend in incidence and mortality from brain cancer observed worldwide in both sexes and most age groups [2]. The incidence of brain and CNS cancers is projected to rise by roughly 40% by 2040 [3]. Previous studies reported that brain cancer is associated with several risk factors, with genetic and environmental factors being the most significant contributors. According to the International Agency for Research on Cancer (IARC), ionizing radiation, such as gamma rays and X-rays, is a recognized risk factor, while potential risks have also been suggested for non-ionizing radiation, including electromagnetic waves, radiofrequency exposure, and cell phone use [2, 4].

Brain cancer, or brain tumors, encompasses a diverse group of diseases caused by abnormal cell growth within the brain. These tumors can be categorized according to their cell of origin, molecular characteristics, and degree of aggressiveness. Primary cancers begin in the brain, while secondary brain tumors are cancers that originate in other organs and subsequently metastasize to the brain. Similar to other cancers, brain tumors may be benign or malignant, with low-grade tumors generally considered non-malignant and high-grade tumors classified as malignant and cancerous [5]. Gliomas, originating from glial cells, are the most prevalent type of brain tumor across all age groups. Among these, glioblastoma is the most aggressive form, representing the predominant malignant brain cancer in adults and accounting for the highest mortality from brain cancer [6].

Currently, numerous conventional treatment options are available to treat brain cancer through removing the tumor mass or inhibiting the tumor growth, including surgery, radiotherapy, and chemotherapy. Surgery is a primary treatment option for solid brain tumors involving the removal of the skull portion. When surgery is not an option, radiotherapy will be done to destroy the tumor cells and shrink brain tumors through the use of high-energy rays. Chemotherapy, on the other hand, uses drugs to destroy cancer cells or slow their growth. However, in most cases, due to their non-specificity, chemotherapeutic agents often kill the normal cells along with the cancerous cells, leading to off-target drug exposure [7]. Malignant tumors or particularly glioblastoma multiforme (GBM), continue to be highly challenging to treat, exhibiting strong treatment resistance and a high likelihood of recurrence [8]. Another current therapeutic hurdle includes incomplete surgical resection, due to the invasive nature of glioblastoma, and therapeutic resistance, due to refractory cells in the heterogeneous glioblastoma cell population [9]. Moreover, these conventional treatments only extend the patient’s survival by 4 to 6 months in most tumor types, including brain cancer, underscoring the need for new therapeutic approaches [10].

Novel strategies, such as immunotherapy, have emerged as safer and less toxic options, as they work by leveraging the body’s own immune system to induce both humoral and cytotoxic immune responses that target and kill cancer cells [11]. There are several types of immunotherapies, such as immune checkpoint inhibitors (ICIs), T-cell-based therapies, and oncolytic viruses. Among these, cancer vaccines have gained increasing attention recently, encompassing cell-based vaccines, peptide vaccines, and nucleic acid-based platforms such as DNA and RNA vaccines. Owing to their ability to stimulate durable and potent immune responses, cancer vaccines hold promise for prophylactic and therapeutic applications, offering a strategy not only to treat tumors but also to reduce the risk of recurrence. Despite the promising potential, the significant limitation of their clinical translation is the immunosuppressive character of the brain along with the blood-brain barrier (BBB) and heterogeneity [12].

The BBB remains the major limitation for all current brain tumor treatments. It functions as a barrier that separates the peripheral circulation from the CNS, which controls and limits the movement of molecules from the systemic circulation and inherently restricts immune responses in the brain, allowing only microglia to mediate immune activity [10]. Immunotherapy offers a promising approach by enabling peripheral immune cells to cross the BBB and mount inflammatory responses against tumors; however, its effectiveness is still constrained by the barrier and the immunosuppressive tumor microenvironment (TME) [13]. Although the leaky BBB/BTB may appear to facilitate drug delivery and improve therapeutic efficacy in glioblastoma, growing evidence shows that the disruption is highly heterogeneous. Because of this variability and the highly invasive nature of glioblastoma, tumor cells often reside in regions with an intact BBB at the tumor margins, where the barrier continues to restrict the distribution of therapeutic agents [14]. To address these limitations, nanoparticle-based delivery systems provide an advanced strategy to enhance BBB penetration and improve the overall efficacy of brain tumor immunotherapy.

Over the last decades, the nanomedicine field, owing to its general properties, such as small size ranging between 1 and 100 nm, enhanced permeability and retention effect (EPR), and high permeability in tumors, has emerged as a promising means in both chemotherapy and gene therapy. It facilitates the targeted delivery of chemotherapeutic drugs and genetic materials across the BBB to brain tumor sites, while reducing damage to healthy tissues [15, 16]. Building on this potential, nanoparticles (NPs) are also being employed to enhance the effectiveness of immunotherapy, offering targeted delivery, improved immune activation, and reduced off-target effects. There are several types of NPs, such as lipid-based NPs, polymeric NPs, and metal-based NPs. To improve the targeting capability, carrier surface modification is employed to include a ligand with a functional group recognizable by receptors on the target cells. This promotes ligand-receptor interactions, allowing the cargoes to be delivered specifically to the intended site [17].

Due to the powerful potential of Artificial Intelligence (AI) computational, when combined with precision-engineered delivery systems offered by nanotechnology, the development of effective immunotherapies can be significantly accelerated. AI algorithms have demonstrated remarkable capabilities in identifying therapeutic targets, predicting immunogenicity and immune responses, and facilitating the design of optimized nanotechnology-based immunotherapies [18]. The combination of AI and nanotechnology enables precise delivery, controlled release, and targeted interaction with immune cells, thereby enhancing the effectiveness of immunotherapies while reducing systemic side effects.

This paper aims to highlight the therapeutic potential of this NP-based strategy in comparison to conventional approaches currently used for brain tumors. It explores various types of NPs and their ability to overcome the challenges of crossing the BBB while harnessing the immune system to selectively target tumor sites, thereby minimizing the risk of off-target effects. Additionally, this review discusses the relevant and ongoing pre-clinical and clinical development of nanotechnology-mediated immunotherapy for glioma through enhanced and targeted delivery of immunotherapeutic agents to lymphoid organs or immune modulators. Furthermore, this review examines the application of AI across multiple stages of immunotherapy development, including predicting immune responses from existing molecular and cellular data, identifying novel therapeutic targets and adjuvants, ensuring safety and optimizing formulation and dosing strategies. By thoroughly evaluating their potential to enhance the efficacy of immunotherapy, this review seeks to pave the way for further advancements in treatment strategies. Ultimately, bridging the gap between nanotechnological immunotherapy innovations and AI-driven clinical applications could contribute to the development of more effective and personalized immunotherapies for brain tumors.

### Current immunotherapy strategies in brain cancer

Surgery, radiotherapy, and chemotherapy remain the primary treatment modalities for brain cancer. However, their effectiveness is often limited by the infiltrative nature of tumors, incomplete surgical resection, restricted access to tumors located in essential brain regions, and the presence of treatment-resistant cell populations that contribute to recurrence [19]. Consequently, patient outcomes remain poor despite aggressive multimodal treatment strategies [20].

To address these challenges, immunotherapy has emerged as a promising approach that harnesses the immune system to recognize and eliminate tumor cells. Preclinical and clinical studies have demonstrated its potential to improve progression-free survival (PFS) and overall survival (OS) compared with conventional therapies [21]. Although BBB protects the central nervous system by restricting the entry of harmful substances, recent studies have indicated that the BBB is frequently disrupted in brain tumors, facilitating the infiltration of immune cells and therapeutic agents into the TME [22, 23]. Furthermore, the discovery of functional lymphatic vessels in the dura mater has challenged the traditional concept of immune privilege in the brain and strengthened the rationale for immunotherapeutic interventions in brain cancers [24].

At the early stages, tumor cells, particularly in brain cancers, can escape detection and destruction by immune cells through various mechanisms including (i) antigenic modulation; (ii) lowering immunogenicity; (iii) immune suppression; (iv) loss of antigen and/or MHC expression; (v) development of defects in antigen processing/presentation; (vi) increased resistance to the cytotoxic effects of immunity (vii) loss of crucial gens involved in the immune response [25, 26]. Due to this series of strategies, cancer immunotherapy aims to strengthen the body’s natural immune defenses to recognize and destroy tumor cells, rather than relying on drugs that directly kill cancer cells as in conventional treatments. Immunotherapeutic approaches work either by inhibiting immunosuppression to release the brakes on anti-tumor immunity or by enhancing immune responses to stimulate anti-tumor activity. However, their effectiveness depends on factors such as the immune characteristics of the tumor, TME, heterogeneity of neoantigen, and tumor mutational burden [21]. Several immunotherapeutic strategies have been explored for the treatment of brain cancer, including immune checkpoint therapy, oncolytic virotherapy, adoptive cell therapy, and cancer vaccines, each possessing distinct advantages and limitations, as summarized in Table 1.

**Table 1:** Summary of the advantages and limitations of various immunotherapy approaches

Immunotherapy	Advantages	Limitations	Ref.
ICIs	• Specifically target immune checkpoints	• Limited by immune resistance • Immune-related adverse effects	[27, 28]
Oncolytic Virus Therapy	• Selectively infect and lyse tumor cells	• Limited by metastatic and recurrent tumor cells • Insufficient delivery of viruses to target cells	[29, 30]
ACT	• Long residence time and proliferative abilities • Targeted approach	• Severe side effects such as cytokine release syndrome • Limited by intratumoral heterogeneity	[31, 32]
Cytokine therapy	• Activation of various immune cells	• Pleiotropic properties • Instability • Off-target effects	[33]
Vaccine	• Precision and specificity • Induction of broad immune responses • Flexibility and personalization	• Weak immunogenicity • Risk of autoimmune reaction	[34–36]

### Immune checkpoint therapy

Immune checkpoints are regulatory pathways that maintain immune homeostasis by balancing stimulatory and inhibitory signals [37]. Tumor cells exploit these pathways to evade immune surveillance, leading to the development of immune checkpoint therapy, which utilizes immune checkpoint inhibitors (ICIs), primarily monoclonal antibodies (mAbs), to restore antitumor immune responses [10].

Among the various checkpoint molecules identified such as lymphocyte-activation gene 3 (LAG-3) and T-cell immunoglobulin and mucin-domain containing-3 (TIM-3), T-cell immunoreceptor with Ig and ITIM Domains (TIGIT), Indoleamine 2,3-Dioxygenase 1 (IDO1), and V-Domain Ig Suppressor of T Cell Activation (VISTA), programmed cell death protein 1 (PD-1), programmed death ligand 1 (PD-L1), and cytotoxic T-lymphocyte-associated protein 4 (CTLA-4) are the most extensively studied and clinically targeted [38, 39]. In line with these discoveries, there are several Food and Drug Administration (FDA)- approved ICIs. These approved monoclonal antibodies include (i) PD-1 inhibitors: Nivolumab, Pembrolizumab, and Cemiplimab; (ii) PD-L1 inhibitors: Atezolimumab, Durvalumab, and Avelumab, and CTLA-4 inhibitor: Ipilimumab [40]. These mAbs have shown promising clinical efficacy in other cancers and are currently being evaluated in clinical trials for glioma.

**Fig. 1:**
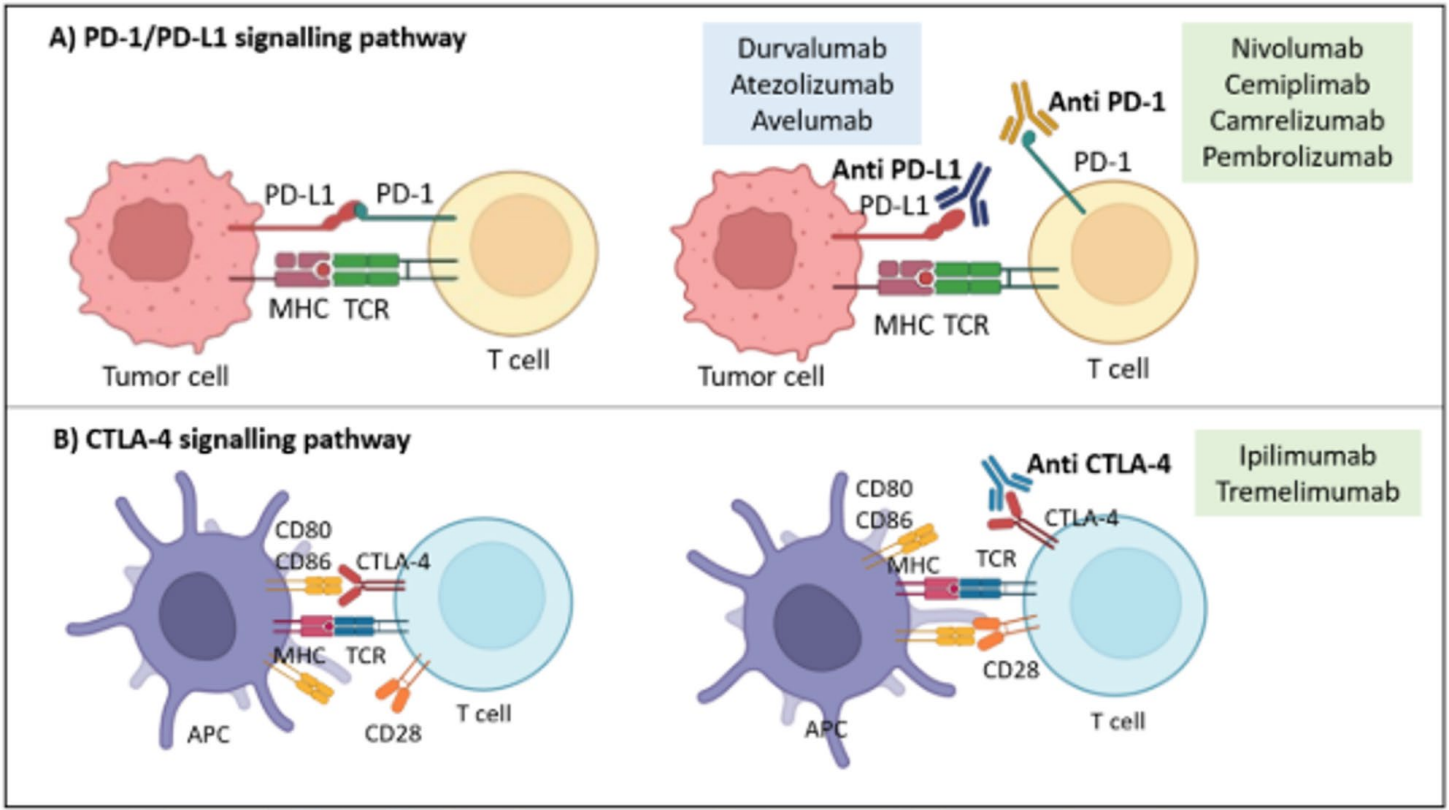
**A **PD-1/PD-L1 signaling pathway and its inhibition. Under normal conditions, when the TCR binds to the antigen presented by tumor cells, the interaction between PD-L1 and PD-1 suppresses T-cell activation, enabling tumor cells to escape immune-mediated destruction. The introduction of ICIs, such as anti-PD-1 or anti-PD-L1 antibodies, prevents this interaction, thereby reactivating T cells to enable the immune system to destroy tumor cells. **B **CTLA-4 signalling pathway and its inhibition. Under normal conditions, when TCR binds to antigen presented by MHC proteins on the APC and CTLA-4 competes with CD28 for binding to CD80/86, leading to suppression of T cell activation. In the presence of an immune checkpoint inhibitor, CTLA-4 binding is blocked, allowing CD28 to interact with CD80/CD86, thereby promoting T cell activation and antitumor immune responses

CTLA-4 is an inhibitory receptor expressed on T cells that negatively regulates T-cell activation by competing with CD28 for binding to CD80 and CD86 on APCs [41, 42]. Through this mechanism, CTLA-4 acts as an important regulator of immune tolerance and prevents excessive immune activation [42]. Consequently, blockade of CTLA-4 can enhance T-cell priming and activation, resulting in improved antitumor immune responses [43]. The mechanism of CTLA-4 signalling and its inhibition is illustrated in Fig. 1. For anti-CTLA-4 therapy, the mAb ipilimumab has entered clinical evaluation in NCT03233152 (Phase I) and NCT02311920 (Phase II) clinical trials.

Another major immune checkpoint pathway involves PD-1 and its ligands, PD-L1 and PD-L2. PD-1 is expressed on activated immune cells, whereas PD-L1 is frequently overexpressed on tumor cells and within the TME [44, 45]. Interaction between PD-1 and PD-L1 suppresses T-cell proliferation, cytokine production and cytotoxic activity, thereby promoting tumor immune evasion [46]. Blockade of this pathway restores T-cell-mediated antitumor immunity and has become one of the most successful immunotherapeutic strategies in oncology [47]. The mechanism of PD-1/PD-L1 signalling and its inhibition is illustrated in Fig. 1.

Clinical trials of ICIs in brain cancer mainly involve anti-PD-1 and anti-PD-L1 antibodies. For anti-PD-1, CheckMate-143 (NCT02017717, nivolumab, Phase III), CheckMate-498 (NCT02617589, nivolumab, Phase III), CheckMate-548 (NCT02667587, nivolumab, Phase III), KEYNOTE-028 (NCT02054806, pembrolizumab, Phase I/II), and NCT02337491 (pembrolizumab, Phase II) have been conducted. In some instances, clinical trials are evaluating combinations of anti-PD-1 therapy with other treatments. For example, the phase II trial NCT04013672 assessed the efficacy of pembrolizumab combined with SurVaxM, a peptide vaccine targeting survivin, a protein commonly produced by cancer cells [48]. For anti-PD-L1, notable studies include NCT02336165 (durvalumab, Phase II), NCT01375842 (atezolizumab, Phase I), and NCT03174197 (atezolizumab, Phase I/II). These studies demonstrated the safety of the approach but were unable to demonstrate a significant survival improvement in the general GBM population.

Beyond checkpoint proteins, mAbs are also employed to target other surface receptors that are abnormally and highly expressed in tumors; for example, EGFR and its mutant form, EGFRvIII, which are frequently overexpressed in GBM cells. Anti-EFGR that have been extensively used in the brain tumors include Depatuxizumab and Nimotuzumab. However, their therapeutic effectiveness remains suboptimal, largely due to the limited ability to cross the BBB and reduced antibody-binding efficacy caused by patient-specific mutations [49].

### Oncolytic virotherapy

**Fig. 2:**
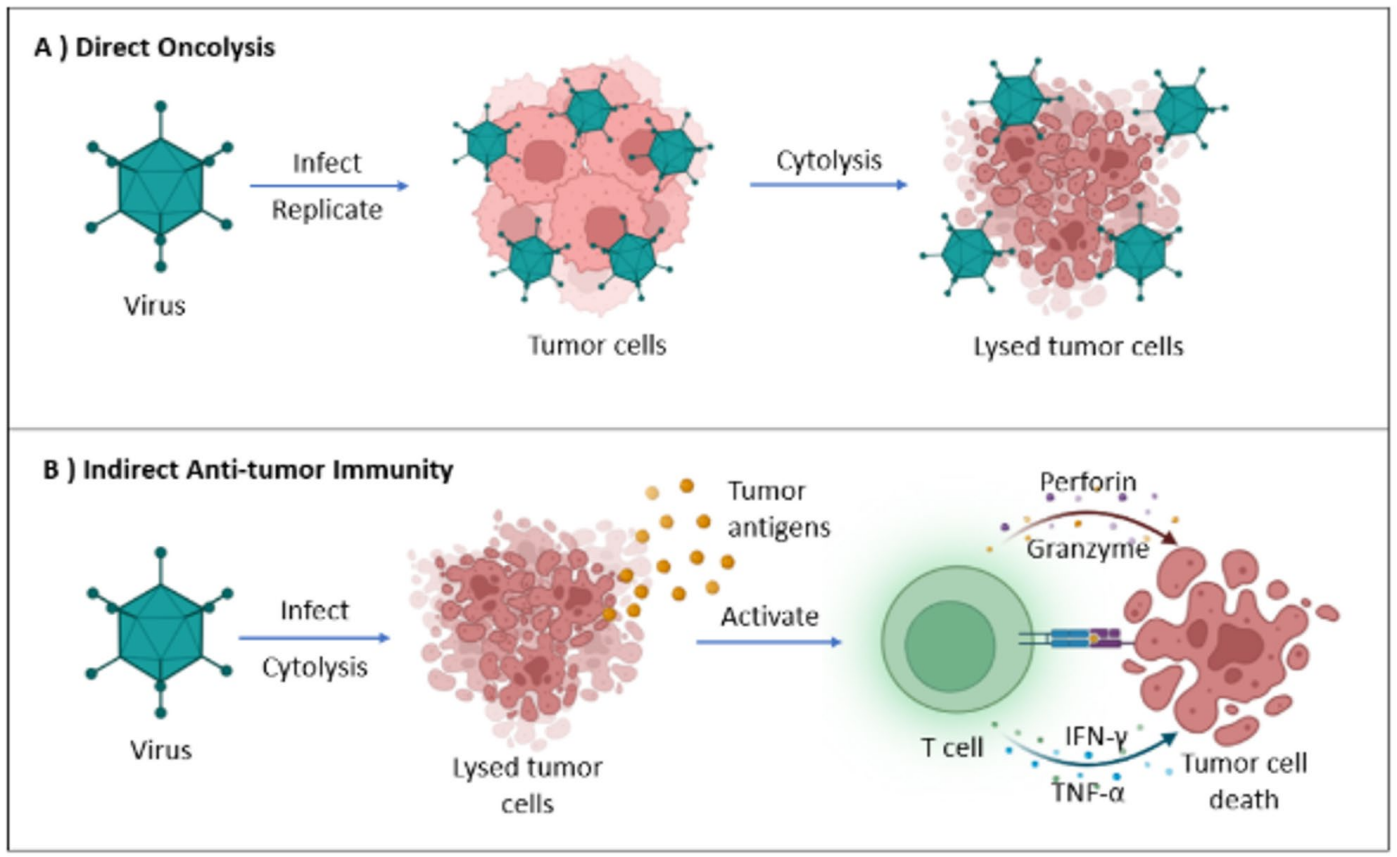
Mechanism of action of oncolytic virotherapy. **A **In direct oncolysis, the oncolytic virus selectively infects and replicates within tumor cells to lyse them. **B **In indirect antitumor immunity, viral infection and subsequent tumor cell lysis release tumor-derived antigens, which activate APCs and stimulate a systemic immune response against tumor cells

Oncolytic virotherapy has emerged as a promising immunotherapeutic strategy for glioblastoma, utilizing replication-competent viruses that selectively infect and destroy tumor cells while simultaneously stimulating antitumor immune responses. These viruses exert therapeutic effects through direct tumor cell lysis, transgene expression, vascular disruption, and immune activation, leading to enhanced recognition of tumor-associated antigens (TAAs) and modulation of the TME (Fig. 2) [50, 51]. Several oncolytic viruses, including Newcastle disease virus (NDV), herpes simplex virus (HSV), reovirus, parvovirus, adenovirus, and poliovirus, have been evaluated for the treatment of brain tumors. Among these, PVSRIPO and DNX-2401 have demonstrated particularly encouraging safety and efficacy profiles in clinical studies involving malignant gliomas [52].

PVSRIPO is a recombinant, non-neurovirulent rhinovirus-poliovirus chimera that targets the poliovirus receptor CD155, a molecule broadly expressed not only on neoplastic cells of solid tumors but also within key components of the TME [53, 54]. Infection with PVSRIPO promotes local inflammation and stimulates innate and adaptive immune responses through activation of macrophages and dendritic cells (DCs), ultimately enhancing tumor cell destruction [55]. Clinical studies, including NCT01491893, have demonstrated acceptable safety and potential survival benefits in patients with recurrent glioblastoma, while ongoing trials are investigating its use in combination with immune checkpoint inhibitors such as pembrolizumab (NCT04479241) [56].

Another extensively investigated candidate is DNX-2401, an oncolytic adenovirus that induces tumor cell lysis and promotes both innate and adaptive antitumor immune responses [57]. In a Phase I clinical trial (NCT00805376), DNX-2401 demonstrated a direct oncolytic effect accompanied by an antitumor immune response; however, it was only partially effective in overcoming T-cell exhaustion [58]. To enhance its therapeutic efficacy, DNX-2401 is currently being evaluated in combination with immune checkpoint inhibitors such as pembrolizumab (NCT02798406), with temozolomide (NCT01956734), and with the cytokine interferon-gamma (IFN-γ) (NCT02197169).

### Adoptive cell therapy

**Fig. 3:**
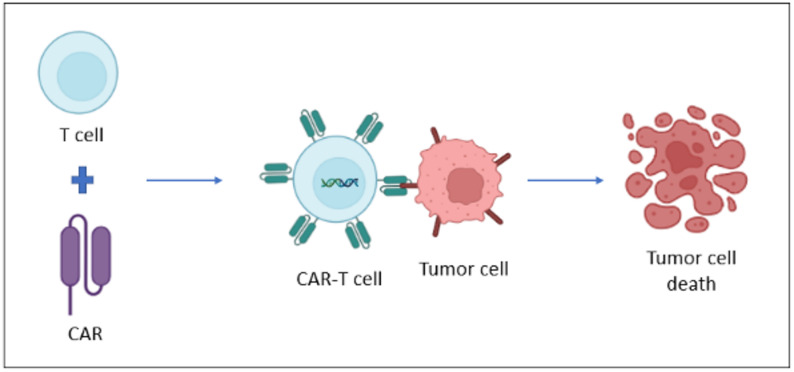
Mechanism of action of CAR-T cell therapy. T cells isolated from the patient are genetically modified to express CARs, which are synthetic receptors designed to recognize specific tumor-associated antigens. Upon administration, these cells bind to target antigens on tumor cells, triggering intracellular signalling cascades that activate the T cells to induce tumor-specific cell death

Adoptive cell therapy (ACT) is an immunotherapeutic approach that involves the isolation, modification, expansion, and reinfusion of immune cells to enhance antitumor responses. The clinical success of ACT in hematological malignancies and melanoma has stimulated growing interest in its application for brain tumors and other central nervous system malignancies. Among ACT strategies, chimeric antigen receptor T-cell (CAR-T) therapy has emerged as one of the most extensively investigated approaches for glioblastoma [59]. CAR-T cells are patient-derived T lymphocytes genetically engineered to express chimeric antigen receptors that recognize TAAs independently. Upon antigen recognition, CAR-T cells become activated and mediate targeted tumor cell destruction (Fig. 3) [60]. The therapeutic efficacy of CAR-T therapy largely depends on the selection of suitable tumor antigens that are highly expressed on cancer cells while exhibiting limited expression in normal tissues [31].

Clinical trials of CAR T-cell therapy for glioblastoma have provided early evidence of its safety and antitumor effectiveness in patients with malignant brain tumors, targeting several common antigens. These include SNC-109 (NCT05868083, NCT06616727), CHM-1101 (NCT05627323), IL-8 CD70 (NCT05353530), B7-H3 (NCT05366179, NCT04385173, NCT04077866, NCT06482905), EGFRvIII (NCT05802693, NCT01454596, NCT02844062), IL13Ralpha2 (NCT04661384) and NKG2D (NCT04717999). Early clinical studies have demonstrated acceptable safety profiles and preliminary evidence of antitumor activity in patients with malignant brain tumors.

For example, EGFRvIII-specific CAR T cells with disrupted PD-1 signaling via CRISPR-Cas9 significantly prolonged survival in orthotopic GBM mouse models [61]. Similarly, CD70-specific CAR T cells demonstrated potent antitumor efficacy against CD70-positive glioblastoma in vitro and in vivo, with minimal toxicity [62]. Furthermore, B7-H3-directed CAR T cells exhibited robust preclinical activity against multiple pediatric brain tumors, including medulloblastoma [63]. Collectively, these findings highlight the potential of CAR T-cell therapy as an evolving strategy for treating GBM and other brain malignancies, although further optimization and advancement are needed to enhance efficacy and overcome the immunosuppressive TME challenges.

### Cancer vaccines for brain cancer

**Fig. 4:**
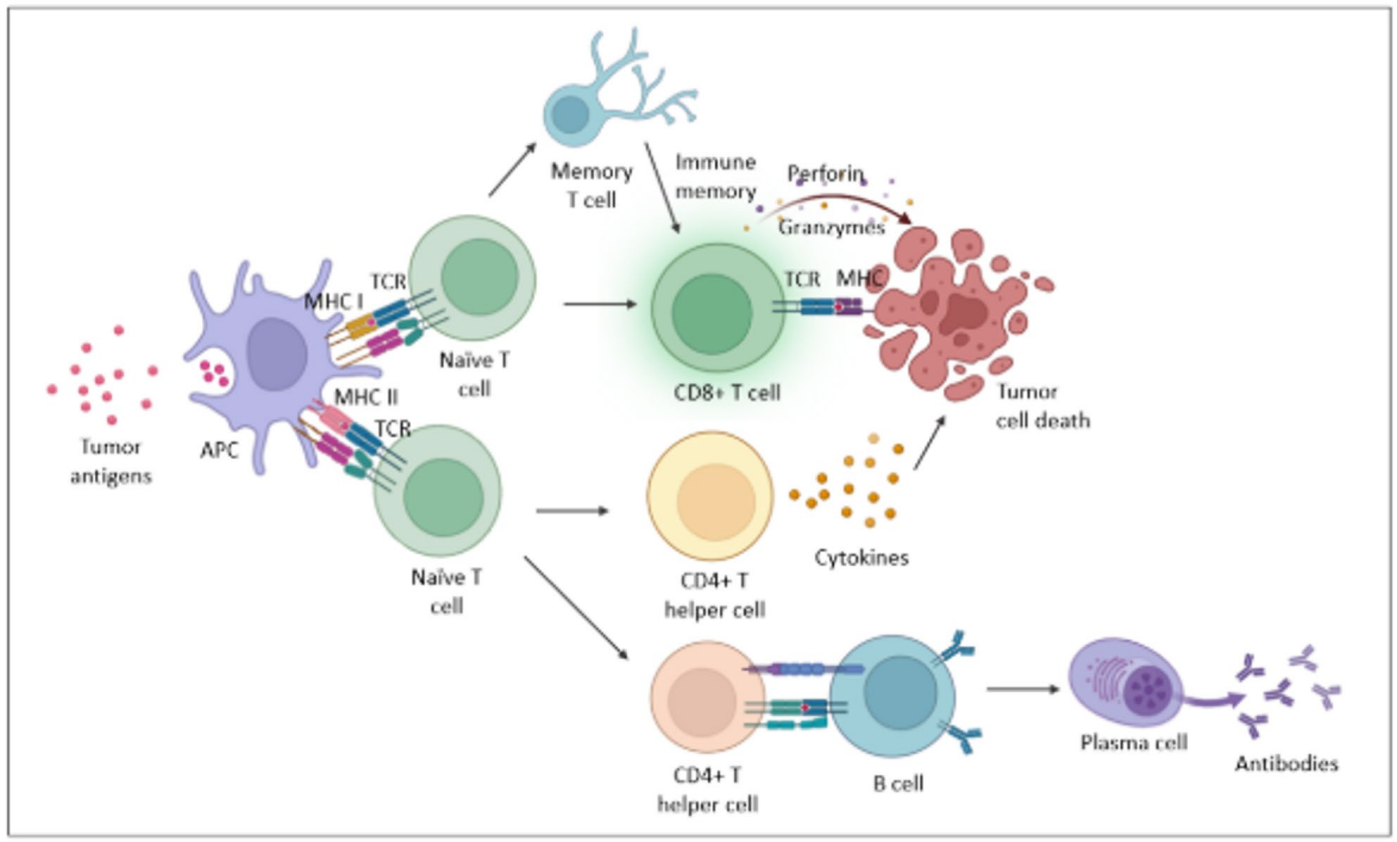
Mechanism of cancer vaccines. Cancer vaccines deliver tumor-derived antigens into the body, where they are recognized, internalized, and processed by APCs. The APCs then present the antigens on MHC molecules, leading to the activation of naïve T cells into cytotoxic CD8 + T cells and CD4 + T helper cells. This activation elicits immune responses that result in tumor cell death and the generation of immune memory, providing long-term protection against tumor recurrence [68]

Cancer vaccines represent an important immunotherapeutic strategy that stimulates the immune system to recognize and eliminate tumor cells by targeting tumor-specific antigens (TSAs), TAAs, or pathogen-derived antigens [64]. Cancer vaccines work by training the immune system to distinguish normal self-antigens from those abnormally expressed on cancer cells, thereby targeting tumors for elimination [65]. Following vaccination, antigens are captured and processed by APCs, which subsequently activate T and B lymphocytes, leading to the generation of antitumor immune responses and long-term immunological memory (Fig. 4) [66]. However, vaccine efficacy remains dependent on factors such as antigen immunogenicity, tumor antigen expression, host immune suppression, and delivery efficiency [67].

Brain cancer vaccines can be broadly categorized into peptide-, nucleic acid-, and cell-based platforms, including DC vaccines and whole-tumor-cell vaccines [69]. Despite differences in formulation, these approaches share the common goal of promoting the activation and expansion of cytotoxic T lymphocytes (CTLs) capable of recognizing and eliminating malignant cells [70].

The selection of suitable antigens is a critical determinant of vaccine efficacy. TAAs are non-mutant molecules shared between normal tissues and cancers, such as overexpressed or differentiation antigens, whereas TSAs, arising from mutations, are unique to cancer cells and are therefore not subject to central tolerance [71]. As of today, there are dozens of TAAs associated with GBM that have been discovered in therapeutic preclinical studies, such as CTCFL, ACTL8, TERT, WT-1, OIP5, XAGE3, IL-13Rα2, EGFR vIII, IL-4, gp100, survivin, MAGE-1, CD133, TRP-2, AIM-2, HER2, EphA2, and YKL-40 [72]. However, unlike TAAs, TSAs trigger stronger and more selective immune responses, exhibiting high immunogenicity and T-cell affinity while minimizing the potential for systemic inflammation [73]. Examples of TSAs associated with GBM include MMP9 and SLC16A3 [74].

Cancer vaccines offer several advantages, including high antigen specificity, the ability to generate long-term immunological memory, and the potential to target residual or recurrent disease [75]. These advantages position cancer vaccines as an emerging strategy among immunotherapies. Clinical studies have reported promising results in terms of safety, OS, and PFS, as summarized in Table 2. Additionally, ongoing clinical trials continue to evaluate their therapeutic efficacy, safety, and feasibility, as shown in Table 3.

**Table 2:** Spectrum of completed brain cancer vaccine platforms in clinical studies. Retrieved from https://clinicaltrials.gov/

Vaccine platform	Condition	Vaccine name	Adjuvant/Drug/Antibody	Phase/ Status	NCT number	Outcomes
DC	Newly Diagnosed Glioblastoma Multiforme	pp65-shLAMP DC	GM-CSF unpulsed PBMC Saline Td	II Completed	NCT02465268	OS The pp65-shLAMP DC with GM-CSF and Td group had a median OS of 16.9 months; pp65-flLAMP DC with GM-CSF and Td has a median OS of 16.2; Unpulsed PBMC and Saline has a median OS of 20.6. PFS For patients treated with pp65-shLAMP DC with GM-CSF and Td, the median PFS was 6.1 months. For patients treated with pp65-flLAMP DC with GM-CSF and Td, the median PFS was 6.4 months. For the control group, the median PFS was 7.1 months.
DC	Newly Diagnosed DIPG and Other Gliomas	K27M peptide	Nivolumab Temozolomide	I/II Completed	NCT02960230 [76]	Safety Profile No grade-4 adverse events reported. OS At 12 months, the OS was 40% for newly diagnosed DIPG patients and 39% for newly diagnosed glioma patients in stratum B. For patients who had an expansion of H3.3K27M-reactive CD8 + T cells, the median OS was 16.1 months compared with 9.8 months in those without such expansion. DIPG patients with below-median baseline levels of myeloid-derived suppressor cells demonstrated longer OS than those with higher levels.
DC	Newly diagnosed or recurrent glioblastoma	Autologous DCs pulsed with lysate derived from a GBM stem-like cell line	Temozolomide Bevacizumab	I Completed	NCT02010606 [77]	PFS For patients with newly diagnosed GBM, the median PFS was 8.75 months. For patients with recurrent GBM, the median PFS was 3.23 months, with a 6-month PFS rate of 24%. OS For patients with newly diagnosed GBM, the median OS was 20.36 months. For patients with recurrent GBM, the median OS was 11.97 months.
DC	Newly Diagnosed or Recurrent Low-Grade Glioma	Tumor lysate-pulsed autologous DC		II Completed	NCT01635283	OS` Among 5 participants, no one experienced sudden or unexpected death. Immune response: Among 5 participants, the mean anti-tumor immune responses were 1.26 for the CD8 biomarker, 1.35 for the PD-1 biomarker, and 6.76 for the PD-L1 biomarker.
DC	Malignant glioma	autologous tumor lysate-pulsed DC	resiquimod polyICLC	II Completed	NCT01204684 [78]	Safety profile Most of the adverse events experienced were grade 1–2. No serious adverse events (Grade 3–4) were reported. Immune response This combination of ATL-DC vaccination with TLR agonists, particularly poly-ICLC significantly enhances systemic interferon-induced immune responses. OS The poly-ICLC group had a median OS of 52.5 months compared to 7.7 months for placebo and 16.7 months for resiquimod PFS The median PFS for poly-ICLC was 31.4 months, compared to 5.5 months for the placebo and 8.1 months for resiquimod.
	Newly and Recurrent Diagnosed GBM Brain Cancer	DCVax-L	-	III Unknown status	NCT00045968 [79]	OS Patients with nGBM receiving DCVax-L had a median OS of 19.3 months; patients treated with SOC, had a median OS of 16.5 months. Patients with rGBM receiving DCVax-L had a median OS of 13.2 months; patients treated with SOC, had a median OS of 7.8 months.
Peptide	IDH1 Positive Recurrent Grade II Glioma	PEPIDH1M	Tenivac Temozolomide	I Completed	NCT02193347	Safety Profile No unacceptable toxicity reported. Immune Responses 42% of patients have a positive vaccine response after 3 post-surgery vaccines.
Peptide	Progressive Diffuse Glioma	IDH1R132H peptide	Avelumab	I Completed	NCT03893903	No results posted
Peptide	IDH1R132H-mutated Grade III-IV Gliomas	IDH1 Peptide		I Completed	NCT02454634 [80]	Safety profile Most of the adverse events experienced were grade 1–2. Immune response IDH1-vac triggered T-cell responses in 26 of 30 patients and B-cell responses in 28 of 30 patients, spanning multiple HLA alleles. Overall response rate (ORR) The ORR was 84.4% of the safety dataset group, corresponding to 86.7% of the immunogenicity dataset group.
Peptide	Newly diagnosed HLA-A2-positive MGMT-methylated glioblastoma	Multipeptide plus XS15	-	I Completed	NCT04842513	No results posted
Peptide	Diffuse intrinsic pontine gliomas	Histone H3.3-K27M Neoantigen	poly ICLC	I Completed	NCT04749641 [81]	Safety Profile Most of the adverse events experienced were grade 1–2. No significant adverse events reported. Immune Responses Neoantigen-specific T-cell responses were identified, and CD4 + and CD8 + TCR clones targeting the H3.3-K27M mutation were validated.
Cell-based	Pediatric Gliomas	HLA-A2 restricted glioma antigen peptides	Poly-ICLC	I Completed	NCT01130077	No results posted

**Table 3:** Spectrum of active or ongoing brain cancer vaccine platforms in clinical studies. Retrieved from https://clinicaltrials.gov/

Vaccine platform	Vaccine	Adjuvant/ Drug/ Antibody	Diagnosis	Phase/ Status	NCT number	Objectives
DC	TTRNA-DC	GM-CSF Autologous Hematopoietic Stem cells (HSCs)	Recurrent oligodendroglioma	I Active, not recruiting	NCT06254326	To determine the feasibility and safety of administering autologous DC, T cells and hematopoietic stem cells
DC	Autologous DCs pulsed with multiple neoantigen peptides	Temozolomide	Newly diagnosed glioblastoma after surgery	II Active, not recruiting	NCT04968366	To determine the safety and preliminary efficacy of autologous DCs DCs loaded with multiple tumor neoantigen peptides
DC	WT1-targeted DC vaccine	temozolomide	Pediatric Patients With High-grade Glioma or Diffuse Intrinsic Pontine Glioma	I/II Active, not recruiting	NCT04911621	To determine the feasibility and safety of DC vaccine administration in pediatric patients with HGG and DIPG
DC	AV-GBM-1	Autologous monocytes	Newly diagnosed glioblastoma	III Not yet recruiting	NCT05100641	To determine whether the addition of AV-GBM-1, a therapeutic, patient-specific DC vaccine, to standard therapy increases overall survival of patients with a recent diagnosis of primary GBM.
Cell-based	TVI-Brain-1	-	Newly diagnosed MGMT unmethylated glioblastoma	II/III Active, not recruiting	NCT05685004	To assess the survival rate and PFS in patients
Cell-based	Personalized peptides	Poly ICLC	Newly diagnosed glioblastoma	I Active, not recruiting	NCT03223103	To assess dose-limiting toxicities, feasibility of personalized MTA vaccine and overall survival
Peptide	PEPIDH1M	vorasidenib	Recurrent idh1 mutant lower grade gliomas	I Recruiting	NCT05609994	To assess the safety and PFS of the combination of PEPIDH1M vaccine and vorasidenib in adult patients
Peptide	PEP-CMV	Temozolomide	Newly diagnosed pediatric HGG and DIPG and recurrent medulloblastoma	II Recruiting	NCT05096481	To determine 4-month PFS in patients with recurrent medulloblastoma, 1-year Overall Survival in patients with newly diagnosed DIPG and 1-year PFS in patients with newly diagnosed HGG
Peptide	Personalized NeoAntigen Peptides/ NeoVax	Poly-ICLC Pembrolizumab Temozolomide	Newly diagnosed glioblastoma	I Recruting	NCT02287428	To evaluate the safety of an investigational intervention and determine the optimal dosage for use in subsequent studies
Peptide	SVN53-67/M57-KLH Peptide	Montanide ISA 51 VG GM-CFS Temozolomide	Newly diagnosed glioblastoma	II Active, not recruiting	NCT02455557	To evaluate 6-month PFS and OS in patients, determine the safety and tolerability of vaccine therapy combined with temozolomide in treating patients with newly diagnosed glioblastoma
DNA	Personalized neoantigen DNA	-	Pediatric patients with recurrent brain tumors	I Recruiting	NCT03988283	To assess the safety, Tolerability and feasibility of adjuvant personalized neoantigen DNA vaccine
DNA	GNOS-PV01	-	Newly diagnosed, unmethylated glioblastoma	I Active, not recruiting	NCT04015700	To assess the safety, feasibility, and immunogenicity of a personalized neoantigen-based vaccine among patients with newly diagnosed, unmethylated glioblastoma
DNA	Personalized Neoantigen DNA vaccine	Retifanlimab	Newly diagnosed, unmethylated glioblastoma	I Recruiting	NCT05743595	To assess the safety and immunogenicity of a personalized neoantigen-based personalized DNA vaccine combined with PD-1 blockade therapy among patients with newly diagnosed, MGMT promoter unmethylated glioblastoma

### Nucleic acid-based vaccine

Brain cancer vaccines can be broadly categorized into nucleic acid-based, peptide-based, and cell-based platforms. Nucleic acid vaccines, including DNA and mRNA vaccines, deliver genetic material encoding tumor antigens to host cells, enabling in situ antigen expression and immune activation [82–84]. DNA vaccines induce antigen-specific immune responses through intracellular expression of tumor antigens and can additionally stimulate innate immune pathways, whereas mRNA vaccines provide direct cytoplasmic translation of encoded antigens without requiring nuclear entry, resulting in efficient protein expression and favorable safety profiles [85].

### Peptide-based vaccine

Like nucleic acid-based vaccines, peptide vaccines target TAAs to elicit an effective immune response against cancer cells. Peptide vaccines utilize tumor-derived epitopes to stimulate antigen-specific T-cell responses and may be formulated as short peptides, long peptides, or modified peptide constructs to improve immunogenicity and therapeutic efficacy [86]. More recently, multiple antigenic peptides, which incorporate several tumor epitopes within a single construct, have been developed to broaden immune activation, address tumor heterogeneity, and reduce the likelihood of immune escape [34].

### Cell-based vaccine

Cell-based vaccines employ immune cells, tumor cells, or tumor-derived materials as sources of antigens. Among these, DC vaccines have received considerable attention for glioblastoma treatment because of their central role in antigen presentation and T-cell activation [66]. Sipuleucel-T remains the only FDA-approved therapeutic cancer vaccine used to treat prostate cancer patients [87]. Since then, researchers have been actively investigating DC vaccines in clinical trials for many other types of cancer, including gliomas. DCVax-L is one of the autologous DC vaccines under clinical exploration for glioma treatment. Liau et al. reported the results of a phase III clinical trial (NCT00045968), the autologous tumor lysate-loaded DC vaccine DCVax-L, when combined with standard of care in patients with newly diagnosed or recurrent GBM, produced a clinically meaningful and statistically significant improvement in median OS [79, 88]. Similarly, in a phase II clinical trial (NCT02366728), vaccination with a cytomegalovirus (CMV)-specific DC vaccine resulted in exceptionally long-term survival in nearly one-third of glioma patients [89]. Whole-tumor-cell vaccines have also been investigated because they present a broad repertoire of tumor antigens, thereby reducing the risk of tumor immune escape and potentially eliciting more comprehensive antitumor immune responses [90].

Overall, the diverse strategies of immunotherapy, including immune checkpoint therapy, oncolytic virotherapy, adoptive cell therapy, and cancer vaccines, offer distinct mechanisms to harness the immune system against brain tumors. Each approach presents unique strengths and limitations in terms of efficacy, safety and clinical. Both preclinical and clinical studies have demonstrated promising antitumor effects and provided valuable mechanistic insights, supporting their continued development and optimization.

### Challenges in glioblastoma treatment

Despite being an emerging approach for the treatment of brain tumors that is generally less toxic, the efficacy of immunotherapy remains limited. This is largely due to the transport-restrictive properties of the BBB, which hinder efficient drug delivery, along with adaptive resistance. While approaches such as ICIs, antibodies, cytokines, and oncolytic therapies have shown promising results in other cancers, they have not translated into significant improvements in clinical outcomes for glioblastoma, which continues to carry a poor prognosis mainly because therapeutic agents struggle to reach the tumor site effectively.

Cancer vaccines face these same obstacles. Although they hold great potential, they encounter major obstacles because of the immunosuppressive brain TME and the restrictive properties of the BBB. These factors limit the ability of immune cells and vaccine components to penetrate the tumor environment, posing a major barrier to therapeutic efficacy. These barriers include the BBB, intra-brain tissues diffusion on and blood-brain-tumor barrier (BBTB) [69]. These multifaceted challenges highlight the need for a deeper understanding of brain-tumor interactions and transport mechanisms, as this knowledge could guide the development of more effective strategies for enhancing immunotherapy delivery and therapeutic outcomes.

### Blood-brain Barrier (BBB)

The BBB remains one of the key obstacles in glioblastoma therapy, as it restricts the entry of immune cells and therapeutic agents, thereby limiting the effectiveness of systemic immunotherapies. Functioning as a highly selective barrier within the neurovascular unit, the BBB maintains central nervous system homeostasis by tightly regulating the exchange between the peripheral circulation and the brain. While this protects the brain from pathogens and toxins, it also prevents most therapeutic agents from entering, allowing only small lipid-soluble molecules and essential nutrients to cross [10, 91]. Additionally, due to the lack of conventional lymphatic vessels and the low levels of circulating T cells in the brain, it remains unclear how activated peripheral immune cells can cross the BBB and effectively target brain tumors [92].

In brain tumors, the BBB may be disrupted, and this condition is referred to as the BBTB, located among brain tumor cells and microvessels. The vascular characteristics of BTBB differ according to the tumor grade. The BBTB typically encompasses continuous, non-fenestrated capillaries that closely resemble normal brain vasculature in low-grade brain tumors. By contrast, high-grade tumor often exhibits disrupted vasculature, largely driven by elevated vascular endothelial growth factor (VEGF) expression in response to the tumor’s high metabolic demands [93].

The formation of the BBTB increases vascular permeability and active efflux of molecules due to the loss of tight junctions, induced by VEGF secretion from tumor cells. This leaky BBB/BBTB caused many to suggest that the barrier is no longer limiting drug delivery. However, the heterogeneous permeability and perfusion of the BTB often result in suboptimal drug delivery [94]. The resulting inconsistencies in drug accumulation and distribution across different tumor regions underscore the challenges posed by the heterogeneous breakdown of the BBB/BTB [95]. In addition, while fenestrations and gaps within the BTB of malignant gliomas permit the passage of small, low-molecular-weight drugs, they are still too narrow to allow efficient transvascular transport of most NPs [96]. Furthermore, intact regions of the BBB can shield certain areas of the tumor, while the uneven distribution of therapeutics across the brain parenchyma may cause limited efficacy in some areas and toxicity in others [97]. These structural complexities make it especially challenging for advanced therapies such as cancer vaccines and nanomedicines to reach glioblastoma effectively, underscoring the need for strategies that can overcome or bypass both the intact BBB and BTB.

### Tumor heterogeneity

The effectiveness of antigen-targeted immunotherapies is often limited by tumor heterogeneity, which contributes to the initiation, progression, and evolution of glioblastoma [35]. Gliomas display extensive cellular and molecular heterogeneity, often causing loss of antigen expression and promoting tumor relapse. This heterogeneity occurs at two levels: inter-tumor heterogeneity, which reflects population-level differences and forms the basis for molecular classifications of glioblastoma, and intra-tumor heterogeneity, which arises within individual tumors and is marked by diverse cellular compositions and molecular alterations [98]. Because of this complexity, vaccines targeting a single antigen frequently encounter immunoresistance, and similar challenges are observed with CAR-T cell therapy [99, 100]. The presence of diverse tumor subpopulations underlies plasticity, while microenvironmental factors such as hypoxia, acidosis, and reactive oxygen species promote genetic instability, together leading to resistance against multiple therapies [101].

### Immunosuppressive TME

The immunosuppressive TME represents one of the major barriers to successful immunotherapy in glioblastoma. Due to the transport-restrictive properties of the BBB, brain tumors receive limited immune cell infiltration, contributing to their classification as cold tumors. They are typically characterized by low levels of tumor-infiltrating lymphocytes (TILs) [102]. Since immunotherapies rely on the presence and activity of immune cells to exert their effects, this immune exclusion represents a major challenge for achieving therapeutic efficacy.

Beyond low infiltration, the TME itself is strongly immunosuppressive. Tumor cells express inhibitory immune checkpoint molecules such as PD-L1, while immunosuppressive cytokines including transforming growth factor-beta (TGF-β), interleukin-10 (IL-10), and prostaglandin E2 suppress antitumor immune responses [103]. In addition, several immunoregulatory cell populations, including regulatory T cells (Tregs), myeloid-derived suppressor cells (MDSCs), and tumor-associated macrophages (TAMs), contribute to immune evasion and tumor progression. Although glioblastoma tissues often contain infiltrating lymphocytes, including T helper cells, CTLs, and Tregs, CTLs frequently exhibit an exhausted phenotype characterized by the upregulation of inhibitory receptors such as PD-1 and CTLA-4, thereby reducing the effectiveness of ICIs [36, 104].

Among the major immunosuppressive cell populations, MDSCs play a critical role in promoting tumor survival and therapeutic resistance. These immature myeloid cells migrate from the bone marrow to the tumor site, where they suppress CTL activation and proliferation while contributing to the generation of immunosuppressive macrophages and DCs [105, 106]. In addition, the presence of high Treg cell levels and immunosuppressive serum factors additionally restricts T-cell proliferation and activity, further limiting effective immunotherapy in glioma patients [72, 107]. Other than that, TAMs are also the main immunomodulatory cell in TME, further support tumor progression by suppressing CTL-mediated cytotoxicity, promoting angiogenesis, and facilitating tumor metastasis. Given the remarkable heterogeneity and functional plasticity of TAMs, MDSCs, and Tregs, elucidating their precise regulatory mechanisms remains challenging but is essential for improving the efficacy of current immunotherapeutic strategies [108].

### Nanotechnology in brain cancer immunotherapy

**Fig. 5:**
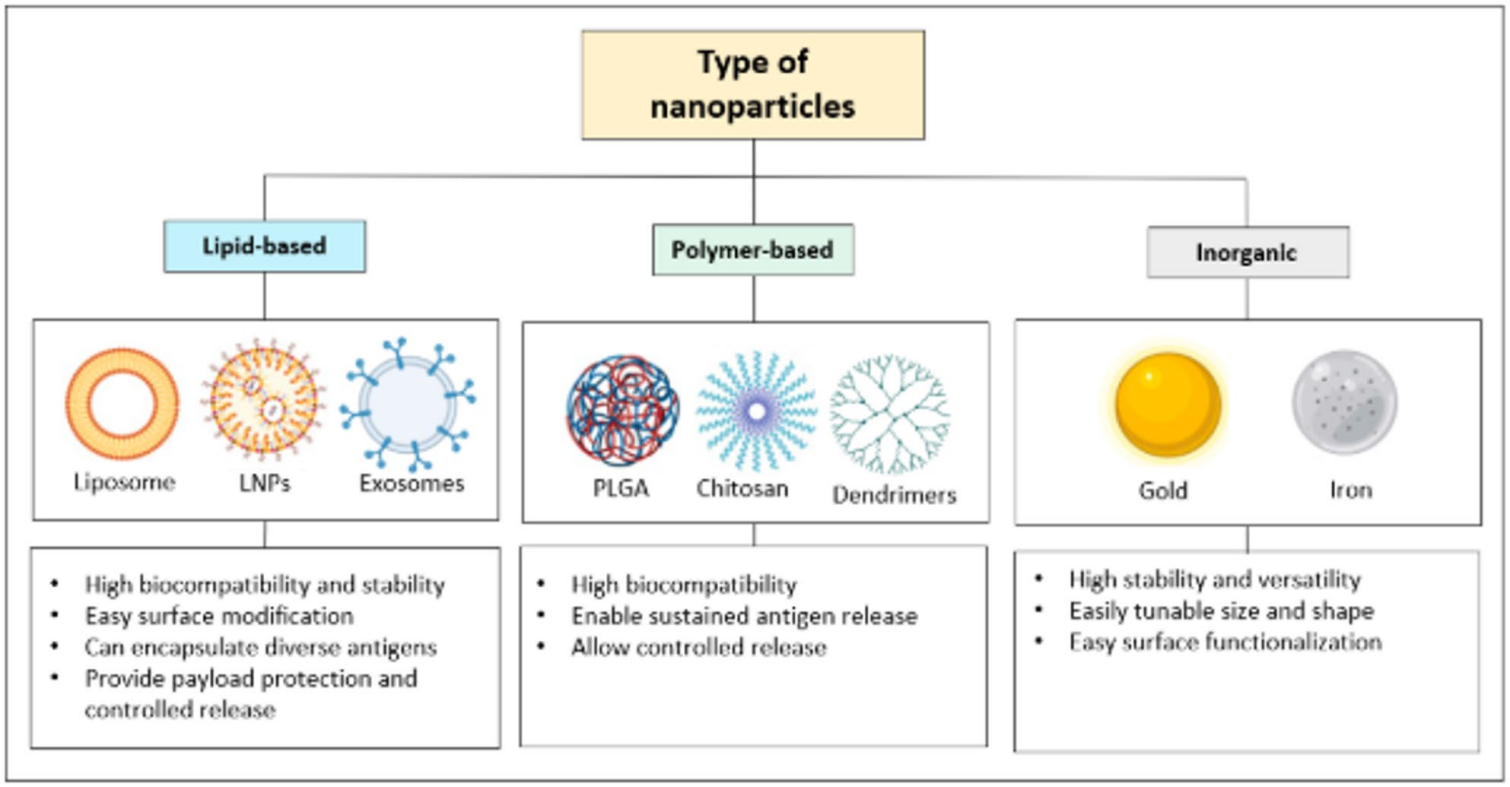
Classification of lipid-based, polymer-based and inorganic nanocarriers and their key advantages for brain cancer immunotherapy applications

To date, extensive research has focused on overcoming the challenges of immunotherapy in glioblastoma. NP-based delivery systems have emerged as versatile platforms capable of transporting a wide range of therapeutic agents across the BBB, providing greater accuracy, stability, and therapeutic impact. In recent years, a wide range of nanocarriers has been designed to overcome the barriers associated with CNS drug delivery, such as lipid-based systems like liposomes, lipid NPs (LNPs), and exosomes; polymer-based NPs such as PLGA; and metal-based platforms, including gold NP (AuNPs) as depicted in Fig. 5. The primary obstacle in delivering drugs and vaccines is the limited access to the tumor site imposed by the BBB and BBTB, which prevents most therapeutic agents from reaching their target with sufficient efficacy. A more comprehensive understanding of the features of these barriers is crucial for improving brain tumor treatment strategies.

Advances in nanotechnology have led to the design of materials and systems capable of protecting therapeutic agents, enhancing their penetration into the CNS, and selectively binding to endothelial receptors at the BBB for controlled release within the brain [109]. By employing passive and/or active strategies to regulate BBB crossing in a controlled, non-invasive manner, NPs have been engineered to deliver a wide range of therapeutics, including small molecules, proteins, genes, and other biopharmaceuticals [110]. The transport routes across the BBB involve using several pathways, such as paracellular and transcellular diffusion, receptor-mediated transcytosis, cell-mediated transcytosis, transporter-mediated transcytosis, and adsorptive-mediated transcytosis [111].

NPs are typically nanosized and possess a high surface-to-mass ratio, which enables efficient therapeutic loading and delivery, establishing them as promising targeted systems that enhance efficacy while limiting side effects [112]. NP-based delivery offers several advantages for brain cancer treatment: (1) the ability to cross the BBB; (2) targeted accumulation at the tumor site; (3) controlled release and improved stability; (4) co-delivery of multiple therapeutic agents; and (5) optimized biodistribution [113, 114]. Several studies have highlighted the efficiency of NP transport across the BBB, along with their ability to selectively deliver immunotherapeutic agents to tumors while minimizing off-target uptake, as summarized in Table 4.

**Table 4:** Preclinical studies employing a nanotechnology-based delivery system for brain cancer immunotherapies

Nanoparticles	Cargoes	Type of Cancer	Significant findings	References
LNP	miR-124	GBM	Enhance antitumor activity, reversing immunosuppression, ability to induce immunological memory; hence protecting against recurrence.	[115]
Liposomes	Tumor-derived RNA	High-grade glioma	Significantly increase the expression of MHC class I/II, B7 co-stimulatory molecules, and maturation markers on APCs, leading to T-cell activation.	[116]
Liposomes	Cy3-labelled RNA or siRNA	Glioma	Effectively deliver mRNA and siRNA to immune cells.	[117]
PLGA	Neutrophil-macrophage hybrid membrane	Glioma	Efficiently cross the BBB and reduce tumor volume.	[118]
PLGA	IL12	GBM	Effectively produce pro-inflammatory cytokines and modulate intracellular levels of pro-inflammatory cytokines.	[119]
PLGA	DC-GL261 fusion cell membrane	GBM	Demonstrated significantly greater accumulation in the spleen and lymph nodes, efficiently penetrated immune organs and activated T cells.	[120]
PLGA	DC Rapamycin	Glioma	Effectively penetrate the BBB in vitro and in vivo, improve the expression of tumor antigens and immune activation and provide long-lasting immunotherapy.	[121]
PLA photobleaching-resistant fluorescent polymer -polylactide copolymers (BPLP-PLAs)	TQM-13-CAR T cell	GBM	Specifically targets and eliminates the brain cancer cells.	[122]
Polymer	Nitric oxide/reactive oxygen species	GBM	Efficiently cross the BBB, transforming the immunosuppressive TME.	[123]
Chitosan	EphrinA1-PE38/GM-CSF	GBM	Increasing the number of DCs activation, effectively killing of glioma tissue and improving the survival of tumor-bearing rats.	[124]
Exosome	CpG	GBM	Efficient cellular uptake in DCs, increase cytokine production, generate antigen-specific immune memory and have long-term tumor-preventive effects.	[125]
PLGA	IL12	GBM	Effectively produce pro-inflammatory cytokines and modulate intracellular levels of pro-inflammatory cytokines.	[119]
PLGA	DC-GL261 fusion cell membrane	Glioma	Demonstrated significantly greater accumulation in the spleen and lymph nodes, efficiently penetrated immune organs and activated T cells.	[120]
PLA photobleaching-resistant fluorescent polymer -polylactide copolymers (BPLP-PLAs)	TQM-13-CAR T cell	GBM	Specifically targets and kills the brain cancer cells.	[122]
PLGA	DC Rapamycin	Glioma	Effectively penetrate the BBB in vitro and in vivo, improve the expression of tumor antigens and immune activation and provide long-lasting immunotherapy.	[121]
Dendrimer	CSF-1R inhibitor BLZ945	GBM	Significantly boosted cytotoxic T-cell infiltration and improved both survival outcomes and markers of immune activation.	[126]
Gold-core silica-shell	ICIs (atezolizumab)	Glioma	Induced the migration of macrophages towards brain tumors, promoted cancer cell apoptosis, significantly enhanced anticancer effects and improved survival rate.	[127]
Gold	Hydrochloroquine (HCQ) Doxorubicin (DOX) anti-PD-L1 antibody	Glioma	Prolonged survival time, increased T cell infiltration, improved anti-glioma immunity and neutralized the immunosuppressive glioma microenvironment.	[128]
Iron oxide	Hsp70 protein	Glioma	Increased the cytotoxic activity of CTLs and survival rate.	[129]

### Lipid-based

As mentioned previously, BBB is the major challenge to treatment efficacy. Hence, one viable strategy to enhance BBB permeability involves increasing the lipophilic properties of therapeutic agents. In line with this, a growing number of studies are focused on improving drug penetration ability by increasing drug liposolubility using lipid-based NPs. Lipid-based nanocarriers such as LNPs, liposomes, and exosomes have been used extensively in the delivery of a wide range of drugs and biomolecules for several applications.

Recently, LNPs have been recognized as a powerful tool for efficient treatment delivery in vivo, inducing the activation of the desired immune response. In recent years, many lipids have been developed, and some have been approved by the FDA [130]. They possess a versatile lipid bilayer structure that enables them to encapsulate therapeutic molecules, particularly nucleic acids, within their core or lipid layers [131]. This design allows for controlled and targeted release, improving therapeutic efficacy while minimizing systemic side effects. Building on these advantages, preclinical studies have explored the potential of LNPs in brain tumor immunotherapy. For example, Yaghi et al. demonstrated that LNPs encapsulating microRNA (miR) could overcome tumor-induced immune suppression and extend survival in a mouse model of glioma [115].

**Fig. 6:**
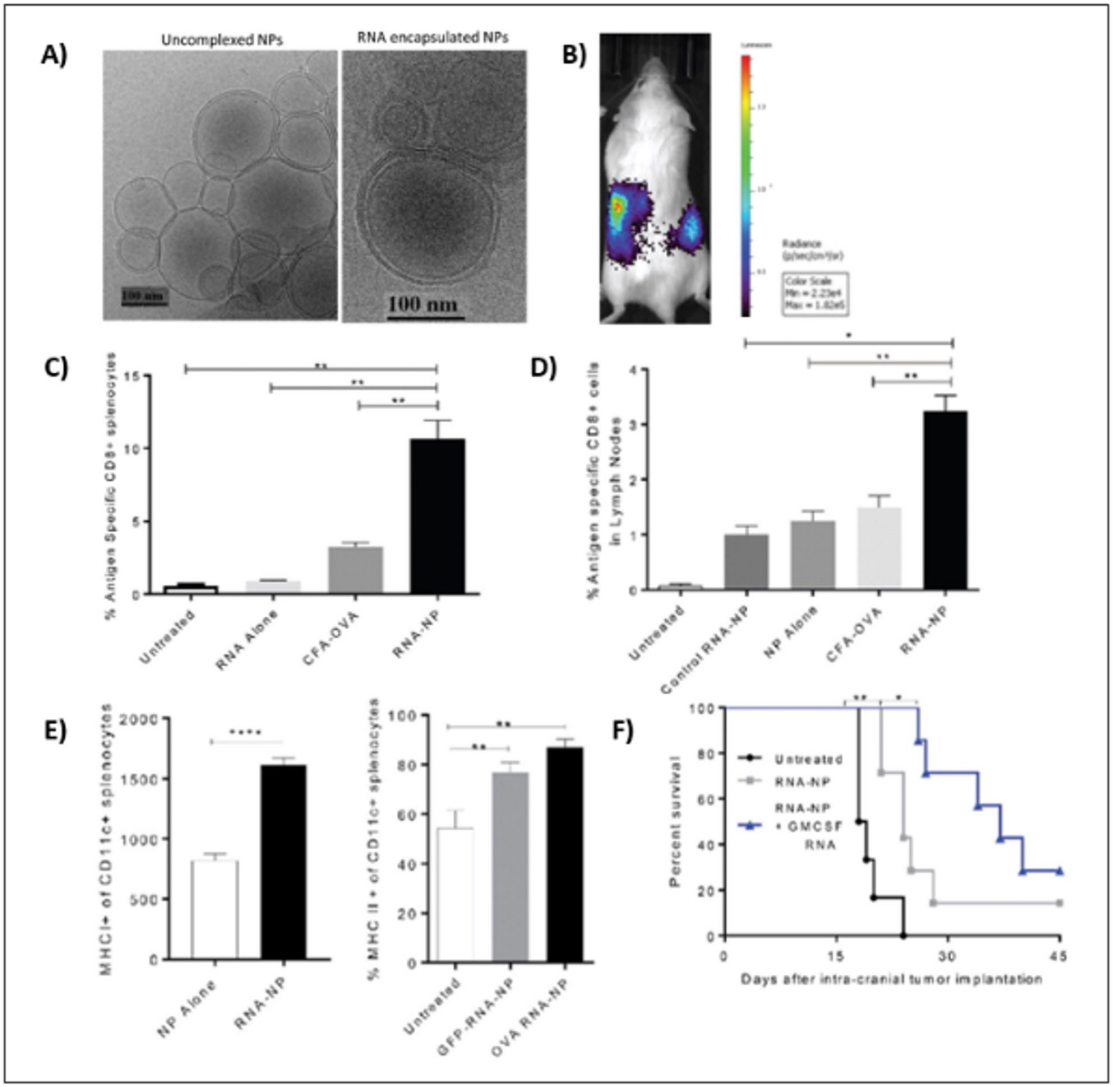
Immunostimulatory and therapeutic effects of RNA-loaded DOTAP nanoparticles against glioma. **A **Cryo-electron microscopy images showing the morphology and size distribution of DOTAP nanoparticles before and after complexation with luciferase mRNA, revealing heterogeneous particles ranging from approximately 70–200 nm. **B **In vivo bioluminescence imaging demonstrating successful translation and expression of luciferase mRNA following administration of RNA-loaded nanoparticles. **C **Comparison of vaccine-induced immune responses showing that OVA RNA-loaded nanoparticles elicited significantly greater expansion of antigen-specific CD8 + T cells than conventional OVA peptide vaccination. **D **Assessment of lymphoid immune responses demonstrating enhanced expansion of antigen-specific CD8 + T cells following vaccination with OVA RNA-loaded nanoparticles compared with control formulations. **E **Systemic immune activation induced by RNA-loaded nanoparticles, evidenced by increased expression of MHC class I and MHC class II molecules on APCs. **F **Therapeutic efficacy of RNA-loaded nanoparticles in an orthotopic glioma model, showing prolonged median survival and increased long-term survival compared with untreated controls [116]

Similar to LNPs, liposomes also have recently been recognized as carriers to reach the CNS due to their therapeutic potential and several advantages, including biocompatibility, high stability, enhanced peripheral circulation, and easy attachment of ligands on the surface of liposomes for a receptor-mediated drug delivery system [132]. Liposomes are spherical lipid vesicles composed of an aqueous core surrounded by phospholipid bilayers. Their amphiphilic nature allows the incorporation of both hydrophobic and hydrophilic drugs [133]. They also provide stabilization of drugs against the external environment and modulated release kinetics of the drug payload [134]. Liposomes have shown promising efficacy as nanocarriers in preclinical models of brain tumors, offering an alternative to traditional cellular immunotherapies. For example, Sayour et al. demonstrated that RNA-loaded liposomes induced strong antitumor responses and potentiated T-cell responses against intracranial B16F10-OVA tumors. The formulated RNA-loaded NPs exhibited a particle size range of approximately 70–200 nm and successfully protected the encapsulated RNA from degradation while facilitating its delivery to antigen-presenting cells [116]. Following administration, the NPs induced efficient in vivo protein expression, promoted the expansion of antigen-specific CD8 + T cells, enhanced the expression of MHC class I and II molecules on APCs, and generated robust systemic immune activation. Importantly, treatment with RNA-loaded NPs significantly prolonged survival in mice bearing intracranial tumors, highlighting their potential as an effective platform for brain cancer immunotherapy (Fig. 6).

Similarly, Grippin et al. created a personalized mRNA-liposome involving DOTAP and cholesterol with the tumor antigens encoding mRNA system, which can effectively fight tumors, remodelling the microenvironment of glioblastoma in the intracranial GL261 tumor model [117]. Incorporating cholesterol into liposomes facilitates the delivery of nucleic acids across the BBB and enhances uptake by myeloid cells associated with certain intracranial tumors.

As a natural nanocarrier, exosomes are generally biodegradable and biocompatible with favorable safety profile because of their low immunogenicity and reduced toxicity [135]. Their ability to transport a diverse array of cargoes, including nucleic acids, lipids, antigens, antibodies, and plasmids, makes them a suitable candidate for immunotherapy-based nanocarriers. Natural exosomes are being collected and bioengineered to enhance their ability to carry therapeutic agents, enhance target selectivity, and prolong their circulation time in the body; and their cargo or contents may vary without significantly changing the natural size or shape of the exosome [136]. Exosomes also exhibit cell-specific targeting capabilities due to their surface proteins that mediate binding to receptors on targeting cells [137]. In addition, their unique lipid composition, including cholesterol and phospholipids, contributes to membrane stability and intercellular signalling functions [138]. Previous studies have demonstrated that exosomes derived from diverse cellular sources, including mesenchymal stem cells, macrophages, DCs, and tumor cells, possess an intrinsic ability to penetrate the BBB [139–141]. Despite their low toxicity, native tumor-derived exosomes may also exert undesirable biological effects by promoting immune evasion, tumor progression, angiogenesis, metastasis, and disease recurrence [142]. Therefore, careful engineering and modification of tumor-derived exosomes are often required to maximize their therapeutic benefits while minimizing potential pro-tumor effects. For example, tumor-derived exosomal miR-7641 has been shown to enhance breast cancer progression and metastatic potential, while exosomal miR-21 contributes to premetastatic niche formation and osteoclast activation, facilitating tumor dissemination to distant organs. These findings highlight that although tumor-derived exosomes possess attractive properties as natural nanocarriers, their intrinsic pro-tumor activities may present safety concerns and therefore require careful engineering before therapeutic application [143].

In addition to serving as carriers, engineered tumor-derived exosomes can be used directly as nanovaccine platforms because they naturally express a broad repertoire of TAAs that can stimulate antitumor immune responses. For instance, Zou et al. demonstrated that CpG-loaded exosome-based nanovaccines significantly activated APCs in lymph nodes and promoted cytokine production, highlighting their effectiveness in eliciting immune activation. Moreover, these nanovaccines induced robust antitumor immune responses in immunosuppressed CT2A-luc glioblastoma mouse models, resulting in markedly improved survival compared to control groups [125]. Similarly, in another study, Li et al. developed a platform based on natural GBM-derived exosomes loaded with CpG for immunotherapy against both primary GBM and recurrent disease. The resulting Exo-CpG formulation demonstrated efficient cellular uptake and effective penetration across the BBB, while eliciting robust and long-lasting antitumor immune responses. These effects significantly inhibited GBM tumor cell proliferation and ultimately prolonged survival in tumor-bearing mice [144].

### Polymer

Biodegradable polymer NPs have been widely investigated for cancer drug delivery and therapy. Their biodegradability provides a less toxic approach, as they can be engineered to degrade in response to biological stimuli, while also enabling controlled and sustained drug release. Common polymer-based NPs explored for targeting brain tumors include chitosan, nanogels, poly(lactic-co-glycolic acid) (PLGA), and dendrimers.

FDA-approved poly(lactic acid) (PLA), PLGA, and their derivatives are the most extensively used polymers in brain drug delivery research due to their biocompatibility and low toxicity. PLGA-based delivery systems can encapsulate a variety of therapeutic agents, including small-molecule drugs, genes, proteins, and vaccines, while protecting them from degradation to ensure high bioavailability [110].

**Fig. 7:**
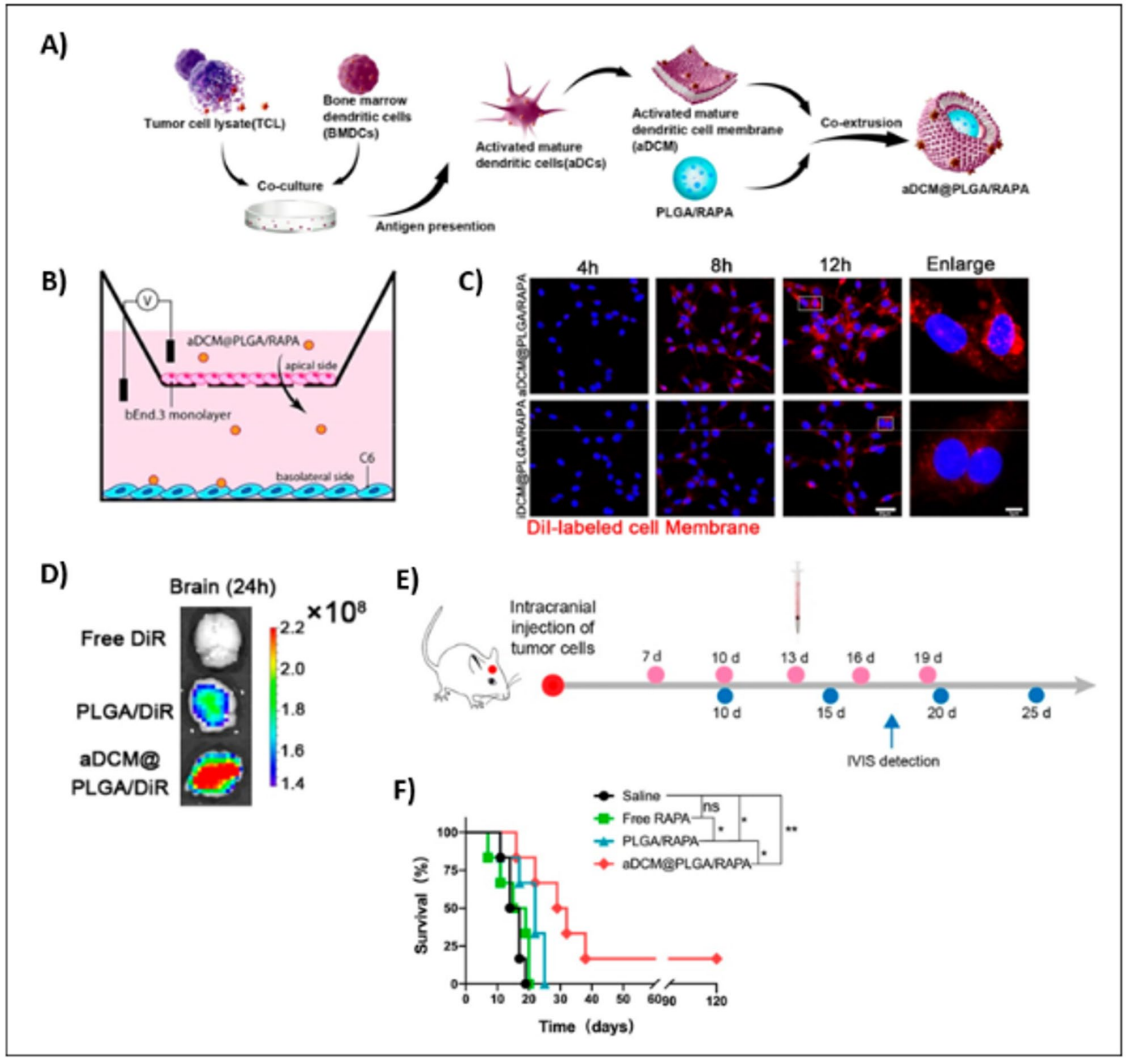
Development and therapeutic evaluation of tumor antigen-activated dendritic cell membrane-coated biomimetic nanoparticles (aDCM@PLGA/RAPA) for glioma immunotherapy. **A **Schematic illustration of the preparation of aDCM@PLGA/RAPA through coating rapamycin-loaded PLGA nanoparticles with tumor antigen-activated dendritic cell membranes. **B **Establishment of an in vitro blood–brain barrier (BBB) model using bEnd.3 endothelial cells and C6 glioma cells to evaluate BBB penetration and tumor-targeting capability. **C **Enhanced cellular uptake of aDCM@PLGA/RAPA in C6 glioma cells after crossing the BBB model, demonstrating superior BBB penetration and homotypic tumor-targeting ability compared with iDCM-coated nanoparticles. **D **In vivo BBB-crossing assessment in an orthotopic glioma model showing significantly higher brain accumulation of aDCM@PLGA/RAPA, with fluorescence intensity greater than free DiR and uncoated PLGA nanoparticles. **E **Evaluation of antitumor efficacy in orthotopic glioma-bearing mice following intravenous administration of saline, free rapamycin (RAPA), PLGA/RAPA, or aDCM@PLGA/RAPA. **F **aDCM@PLGA/RAPA markedly inhibited tumor growth and prolonged survival, increasing median survival to 29 days compared with 14, 15, and 22 days for saline, free RAPA, and PLGA/RAPA groups, respectively. Notably, one treated mouse survived approximately 120 days with almost undetectable tumor signals [121]

In a recent study, Sousa et al. investigated NP-based immunotherapy by encapsulating IL-12, a potent anticancer cytokine, into PLGA NPs. The findings demonstrated that PLGA delivery enhances pro-inflammatory cytokine expression in J774.1 macrophages and GBM cells compared to free IL-12 [119]. Other than that, PLGA-based cancer vaccines also enhance the therapeutic effects. Ma et al. proved this strategy by developing NPs coated with DC-GL261 fusion cell membrane (DGNPs) and evaluating their efficacy in mice with GL261 gliomas. From this study, they found that treatment with DGNPs effectively inhibited tumor development and prolonged the survival of tumor-bearing mice [120]. Similarly, Kim et al. also demonstrated that PLA-based CAR-T cell immunotherapy improved targeted delivery while reducing systemic toxicity [122]. PLGA-based nanovaccines also have the potential to cross the BBB in glioma. For example, Ma et al. evaluated the ability of the nanoplatform strategy to cross the BBB and improve the immune microenvironment using an orthotopic glioma mouse model. This biomimetic design combined the immune-recognition properties of activated DCs with the drug-delivery capability of PLGA NPs. As illustrated in Fig. 7, the resulting aDCM@PLGA/RAPA NPs demonstrated enhanced BBB penetration, increased accumulation within glioma tissue, and superior cellular uptake compared with uncoated NPs. Furthermore, treatment significantly inhibited tumor growth and prolonged survival in orthotopic glioma-bearing mice, highlighting the potential of DC membrane-coated NPs to improve the efficacy of glioma immunotherapy [121].

**Fig. 8:**
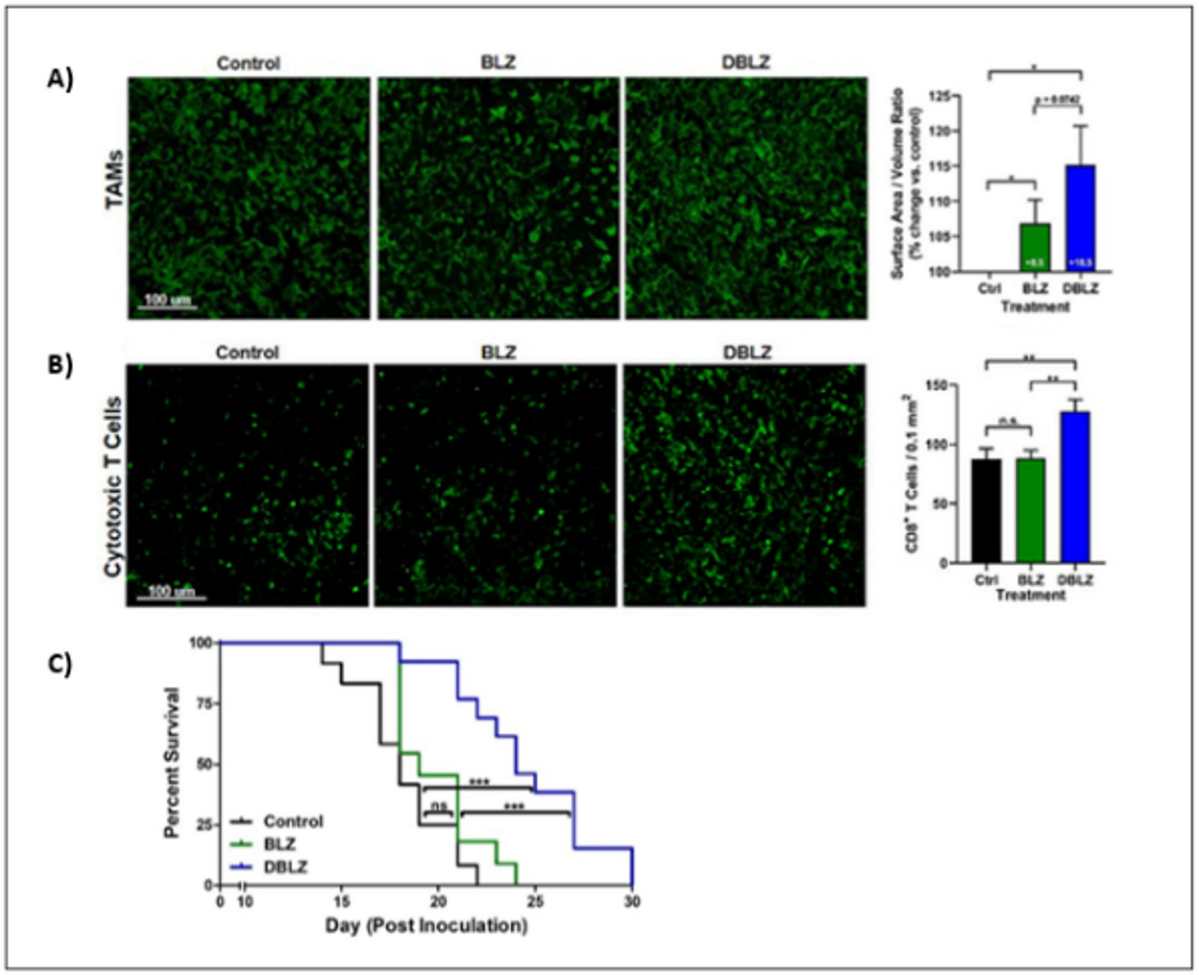
Targeted dendrimer-mediated delivery of the CSF-1R inhibitor BLZ945 enhances antitumor immune responses in glioblastoma. **A **Dendrimer-conjugated BLZ945 (DBLZ) reprogrammed tumor-associated macrophages (TAMs) from a pro-tumor to an anti-tumor phenotype, as indicated by morphological changes following treatment. **B **DBLZ treatment increased the infiltration of cytotoxic CD8 + T cells within the tumor microenvironment, suggesting enhanced antitumor immune activity. **C **A single systemic administration of DBLZ significantly improved survival in orthotopic glioblastoma-bearing mice, prolonging survival by approximately 30% compared with control and free BLZ945-treated groups [126]

Dendrimers are polymer-based drug delivery systems with a highly branched, 3D tree-like structure. Their well-defined design allows controlled drug loading and release [36]. Due to their multifunctional nature, dendrimers serve as effective drug delivery platforms, especially when functionalized with targeting molecules such as peptides or antibodies to direct therapeutics specifically to cancer cells [145]. For example, in a study conducted by Liaw et al., dendrimer-mediated immunotherapy can facilitate a specific tumor immune response, thereby improve therapeutic efficacy while minimize toxicity [126]. Figure 8 highlights a targeted immunomodulatory strategy in which the CSF-1R inhibitor BLZ945 was delivered using dendrimer NPs to selectively target tumor-associated macrophages (TAMs) within glioblastoma. The dendrimer-conjugated formulation (DBLZ) effectively reprogrammed TAMs from a pro-tumor to an anti-tumor phenotype, enhanced cytotoxic CD8 + T-cell infiltration into the TME, and significantly prolonged survival in orthotopic glioblastoma-bearing mice compared with free BLZ945 treatment. These findings demonstrate the potential of dendrimer-based nanocarriers to improve the therapeutic efficacy of immune-modulating agents through targeted delivery to the TME.

Currently, much attention has focused on Dynabeads, which are polymer-based magnetic particles composed of Fe3O4, giving them superparamagnetic properties, and coated with specific antibodies such as anti-CD3 and anti-CD28. These beads have been consistently demonstrated to induce efficient T-cell activation and proliferation [146]. Beyond their immunostimulatory capacity, they offer practical advantages, including ease of handling, low cost, and surface modifiability for the selective expansion of antigen-specific T cells [147].

### Inorganic

This group of NPs refers to elements that have a wide array of physical and structural properties, making them promising candidates for therapeutic delivery. Inorganic NPs encompass a wide variety of materials, including elemental metals like gold, metal oxides such as iron oxide, and various metal salts.

One of the inorganic NPs is gold NPs (AuNPs), which are inherently inert and biocompatible. Their therapeutic properties are strongly influenced by factors such as size, shape, and the nature of these surface ligands, which can be tailored for diverse biomedical applications [113]. The resistance of gold to chemical oxidation minimizes degradation and corrosion, allowing AuNPs to maintain their structural integrity and stability over long periods. These unique physicochemical features make AuNPs highly versatile, enabling their use across a wide spectrum of biomedical applications, including targeted drug delivery for brain diseases [148].

Gold nanomaterials offer several distinct advantages over conventional drug delivery methods for treating brain diseases. Given the disappointing clinical trials combining immunotherapy with radiotherapy in glioblastoma to date, Chen et al. proposed a nanomedicine-assisted radiation strategy to improve the effectiveness of the ICI α-PD-L1. In their approach, AuNPs were used as nanocarriers and further coated with silica dioxide (SiO₂). This study found that the combination of radiation with Au@SiO2 NPs significantly enhanced the therapeutic effects of a-PD-L1 in GL261-brain tumor mice model, leading to improved survival outcomes [127]. Gold NPs conjugated with PD-L1 inhibitors have demonstrated promising outcomes in glioblastoma models, effectively modulating immune responses and leading to a reduction in tumor volume [128]. Shevtsov et al. further demonstrated that magnetic nanocarriers, specifically superparamagnetic iron oxide NPs (SPIONs) coated with heat shock protein 70 (Hsp70), can be effectively used as a nanovaccine to induce an anti-tumor immune response in experimental C6 glioma models. This approach significantly inhibits tumor growth and improves survival rates by enhancing the immune system’s ability to target cancer cells through increased IFN-γ secretion and enhanced T-cell activity and infiltration [129].

### Current clinical trials of nanotechnology-based immunotherapy

Several nanotechnology-based immunotherapeutic strategies, particularly cancer vaccines, have progressed to clinical evaluation for brain cancer, as summarized in Table 5. Relevant clinical trials on brain tumor immunotherapy were identified through a search of the National Institutes of Health Clinical Trials database (ClinicalTrials.gov). Although these studies represent an important step toward clinical translation, progress remains limited, emphasizing the need for further research to establish the safety and efficacy of nano-vaccine-based approaches.

**Table 5:** Clinical trials employing a nanotechnology-based delivery system for brain cancer vaccines. Retrieved from https://clinicaltrials.gov/

Types of Immunotherapies	Nano-carriers	Cargoes	Phase	NCT Number	Tumor types	Outcomes Measures
Vaccine	Lipid	pp65 fl. LAMP mRNA Tumor mRNA	I	NCT06389591	Recurrent GBM	Manufacturing feasibility (up to 20 weeks) Incidence of investigational treatment related toxicities (AEs and SAEs must be reported) (17 months)
	Lipid	pp65 fl. LAMP mRNA Tumor mRNA	I/II	NCT05660408	Recurrent Pediatric High-Grade Glioma	Maximum tolerated dose within 14 months Feasibility of generating patient-specific pp65/tumor mRNA RNA-LP within 14 months
	Lipid	pp65 fl. LAMP mRNA Tumor mRNA	I	NCT04573140	Adult GBM Pediatric High-Grade Glioma	Manufacturing feasibility (up to 20 weeks) Safety of the vaccine (up until 2 weeks after the 3rd vaccine Maximum Tolerated Dose (up to 30 months)

### Rationale for nanotechnology-based immunotherapy for brain cancer

The BBB is a natural protective membrane that acts primarily as a barrier, restricting the penetration of biological substances or large molecules into the brain. Consequently, most therapeutic agents struggle to cross the BBB because of their large molecular size. NPs provide several advantages in this context, as their design can be tailored based on size, surface properties, and the specific type of therapeutic payload they carry [149]. Optimizing NP size is crucial to achieving an effective balance between permeability through the BBB and retention at the tumor site [150]. Moreover, their physiochemical properties, such as high drug loading, prolonged blood circulation time, enhanced stability, facile surface modification for active targeting, and controlled drug release, also help to enhance the delivery efficiency of immunotherapeutic agents to brain tumors [151].

To further enhance transport across the BBB and accumulation within brain tumors, surface functionalization has emerged as a key strategy [152]. Nanocarriers can be modified with targeting ligands such as antibodies, peptides or small molecules that recognize receptors overexpressed on BBB endothelial cells or tumor cells [153]. These modifications help to promote receptor-mediated transcytosis, enhance pharmacokinetic behavior and facilitate deeper tissue penetration [151].

For instance, the transferrin receptor is one of the most commonly targeted receptors in brain cancer [154]. Conjugating transferrin or transferrin-mimicking ligands onto the NP surface enables their interaction with the transferrin receptor, facilitating receptor-mediated transcytosis across the BBB and enhancing accumulation at the tumor site [155]. In addition, receptors such as the epidermal growth factor receptor (EGFR) are also frequently overexpressed in brain tumors and play a pivotal role in tumor progression [156]. Liu et al. demonstrated that dendrimers functionalized with dual-targeting ligands, which are angiopep-2 and an EGFR-binding peptide, exhibited BBB permeability and glioma-targeting efficiency through receptor-related protein 1 (LRP1)- and EGFR-mediated transcytosis and resulted in prolonged survival and reduced systemic toxicity in glioma-bearing mice [157].

Beyond improving drug delivery, nanotechnology also enables combination immunotherapy approaches through the co-delivery of multiple therapeutic agents within a single platform. Such strategies allow the simultaneous modulation of different immune pathways and may enhance antitumor efficacy. For example, a previous study demonstrates that bi-adjuvant/neoAg co-delivering nano-vaccines, particularly when combined with immune checkpoint blockade, offer a promising strategy for robust antitumor immunity against aggressive brain tumors like glioblastoma, with a favourable safety profile [158]. Collectively, these findings highlight that nanotechnology-based immunotherapeutic platforms not only enhance drug delivery and targeting efficiency but also open new avenues for synergistic and effective treatment strategies against brain cancer.

Importantly, nanotechnology-based immunotherapy is not limited to improving therapeutic delivery but can also directly modulate the immunosuppressive TME. As discussed previously, tumor-related immunosuppressive cells including Treg cells, TAMs and MDSCs play central roles in promoting immune evasion and suppressing antitumor T-cell responses. Consequently, engineered NPs have increasingly been explored as tools to selectively target and reprogram these cell populations. For example, Wang et al. developed microenvironment-responsive polymeric NPs with IL-2 payload to re-educate TAMs from a tumor-supportive phenotype to a tumor-suppressive phenotype with minimal toxicity [159]. Similarly, Li et al. designed neutrophil membrane-coated PLGA NPs to suppress the expansion and tumor trafficking of MDSCs, thereby alleviating immunosuppression within the TME [160]. Furthermore, Qu et al. developed peptide-conjugated hybrid NPs for targeting Treg cells in TME, resulting in reduced intratumoral Treg accumulation, enhanced antitumor immunity, prolonged survival and significant tumor inhibition [161]. Collectively, these studies demonstrate that nanotechnology-based immunotherapy can simultaneously enhance therapeutic delivery and actively remodel the immunosuppressive TME, providing a strong rationale for its application in brain cancer treatment.

### Nanotechnology-based immunotherapy clinical translation challenges and future perspectives

Despite significant preclinical advancements in using nanocarriers to deliver immunotherapeutic agents for brain tumors, their translation into clinical application remains challenging. Until today, numerous NP platforms, ranging from liposomes, polymers, and inorganic materials, have demonstrated efficient BBB penetration, tumor targeting, and therapeutic efficacy in animal studies. However, only a few have progressed to clinical trials due to several significant challenges. Although certain nanocarriers, like liposomes and PLGA, have been approved by the FDA for various therapeutic applications, several obstacles continue to hinder their successful clinical translation for brain cancer. Key challenges include BBB and tumor heterogeneity, large-scale manufacturing and reproducibility, safety and long-term toxicity concerns and regulatory requirements.

BBB heterogeneity and the highly heterogeneous nature of brain tumors remain major barriers to the clinical translation of nanotechnology-based immunotherapies [162]. Customized vaccines designed to target tumor-derived antigen profiles are increasingly favored for their advanced technological design, potent immune stimulation, and lower risk of immune evasion caused by tumor mutations. Considering the extensive heterogeneity of tumors, which can result in the survival and expansion of treatment-resistant subpopulations, effective nanocarrier strategies should be designed to simultaneously target multiple tumor cell populations. This can be achieved through the incorporation of ligands that recognize receptors overexpressed across different tumor regions and cellular subtypes [163]. Furthermore, integrating these nanocarrier-based approaches with immunotherapeutic or conventional treatment modalities may help overcome the immunosuppressive TME, thereby enhancing therapeutic efficacy and improving overall treatment outcomes [86].

Another key barrier to clinical translation lies in the manufacturing and scalability of nanomedicine-based immunotherapies. Although these therapies show great potential, issues such as production efficiency, standardization, and reproducibility continue to hinder progress. The current manufacturing process is complex, time-consuming, and costly, making it difficult to ensure consistency across batches. Even small variations in NPs size, surface charge, or drug loading can influence pharmacokinetics and therapeutic outcomes [109].

**Fig. 9:**
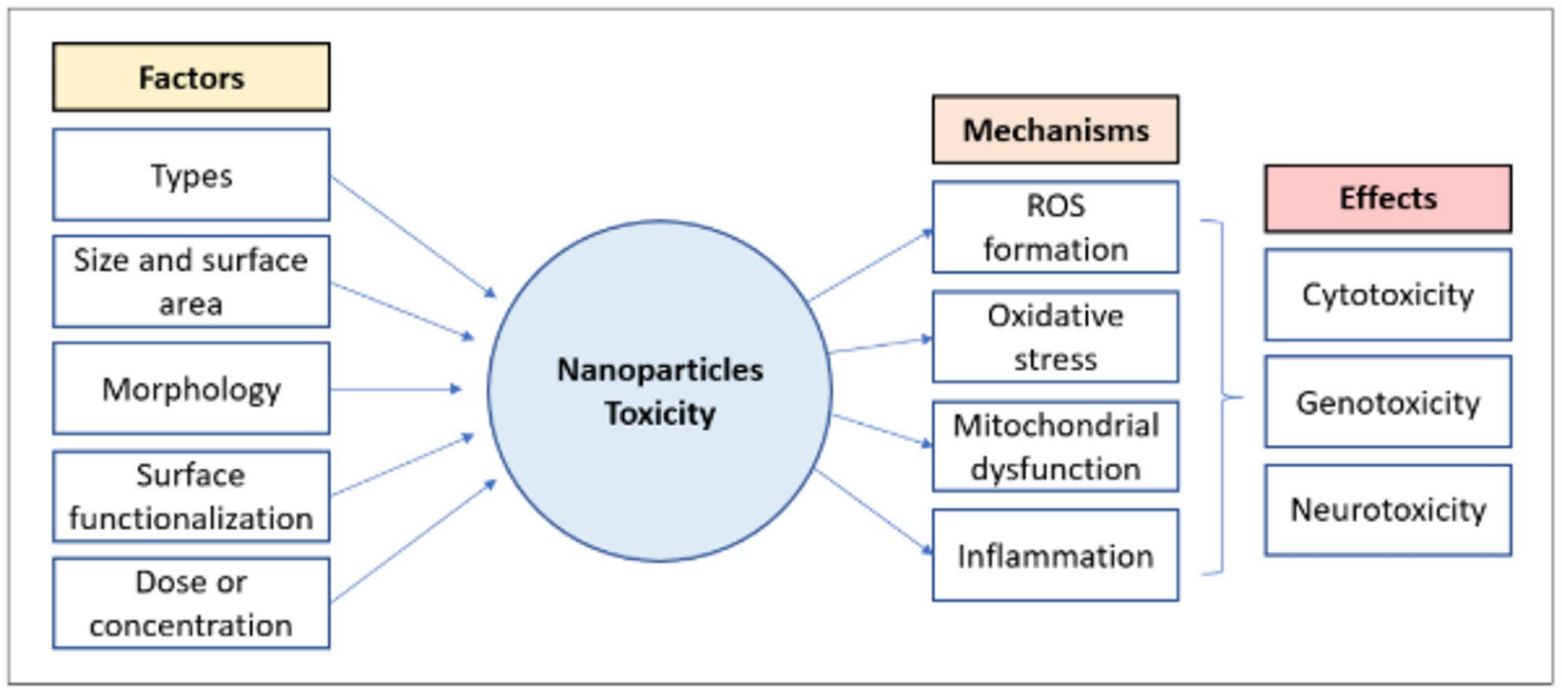
Factors affecting NP toxicity and the associated biological effects. NP toxicity is influenced by physicochemical characteristics including composition, size, surface area, morphology, surface functionalization and administered dose. These properties affect NP interactions with biological systems and may induce ROS generation, oxidative stress, inflammation, and cellular dysfunction, ultimately resulting in cytotoxicity, genotoxicity, neurotoxicity and other adverse biological effects

Safety remains another critical consideration for the clinical translation of nanotechnology-based immunotherapies. In peripheral organs, nanomaterials are typically cleared via renal filtration or liver pathways; however, their long-term fate following accumulation within the central nervous system remains incompletely understood [164]. Although NPs offer significant therapeutic advantages, they may also pose health risks due to their ability to cross biological barriers, accumulate in tissues, and interact with biological systems in unintended ways [165–167]. Their toxicity is influenced by multiple physicochemical properties, including composition, size, surface area, morphology, surface chemistry, and administered dose [168]. Depending on the duration of exposure, NP-associated toxicity can be broadly categorized into acute and chronic toxicity. Acute toxicity includes immediate adverse effects such as inflammation, oxidative stress and mitochondrial dysfunction, whereas chronic toxicity may result in long-term organ damage, neurotoxicity, cytotoxicity and genotoxicity [169–171]. Furthermore, because NPs are specifically designed to cross the BBB and accumulate within the brain, understanding their long-term neurotoxicity and clearance from the central nervous system is particularly important. The major factors affecting NP toxicity and the resulting biological consequences are summarized in Fig. 9.

Moreover, the biodistribution and retention of NPs in the body pose important safety concerns. Long-term accumulation of poorly degradable nanomaterials may induce persistent biochemical and immunological responses, leading to chronic inflammation and tissue damage [171, 172]. Furthermore, NP aggregation in biological environments can alter biodistribution and increase the risk of vascular obstruction or embolic events as reported in many previous studies [173, 174]. NPs may also trigger excessive cytokine production and non-specific immune activation, potentially resulting in cytokine release syndrome [175]. Although such immune activation is generally undesirable, controlled stimulation of immune responses may be beneficial in cancer immunotherapy. Therefore, optimizing NP biodegradability, stability and immunocompatibility remains essential for ensuring their long-term safety and clinical applicability.

From a regulatory perspective, the clinical translation of nanomedicines is further complicated by the need for comprehensive physicochemical characterization, stability assessment, and strict adherence to Good Manufacturing Practice (GMP) standards [176]. Safety and toxicity considerations also present significant regulatory challenges because the unique biological interactions and potential long-term effects of nanomaterials often require more extensive evaluation than conventional therapeutics. Consequently, the FDA currently assesses nanotechnology products on a case-by-case basis using the combination product framework [177]. However, concerns regarding inconsistent product classification and the adequacy of existing regulatory pathways for evaluating nanomaterial safety have led to calls for more standardized regulatory guidelines.

To overcome these challenges, the integration of artificial intelligence (AI) and advanced computational modelling is emerging as a powerful tool to optimize NP design, predict biological interactions, and support the development of more personalized, safe and efficient formulations [178]. AI can also assist in identifying suitable therapeutic targets and bioactive compounds by analyzing complex biological datasets, improving precision and efficiency in drug development [179, 180]. Furthermore, AI enables rapid screening and optimization of large numbers of molecular structures with high predictive accuracy, significantly reducing development time and resource requirements [181].

In light of these challenges, future research should focus not only to improve the design of nanocarriers and enhance their therapeutic effectiveness, but also addressing manufacturing, safety and regulatory considerations. Paying attention to these aspects is crucial for bringing nanotechnology-based immunotherapies successfully from laboratory to clinical practice.

### Application of Artificial Intelligence (AI) in immunotherapy for brain cancer

**Fig. 10:**
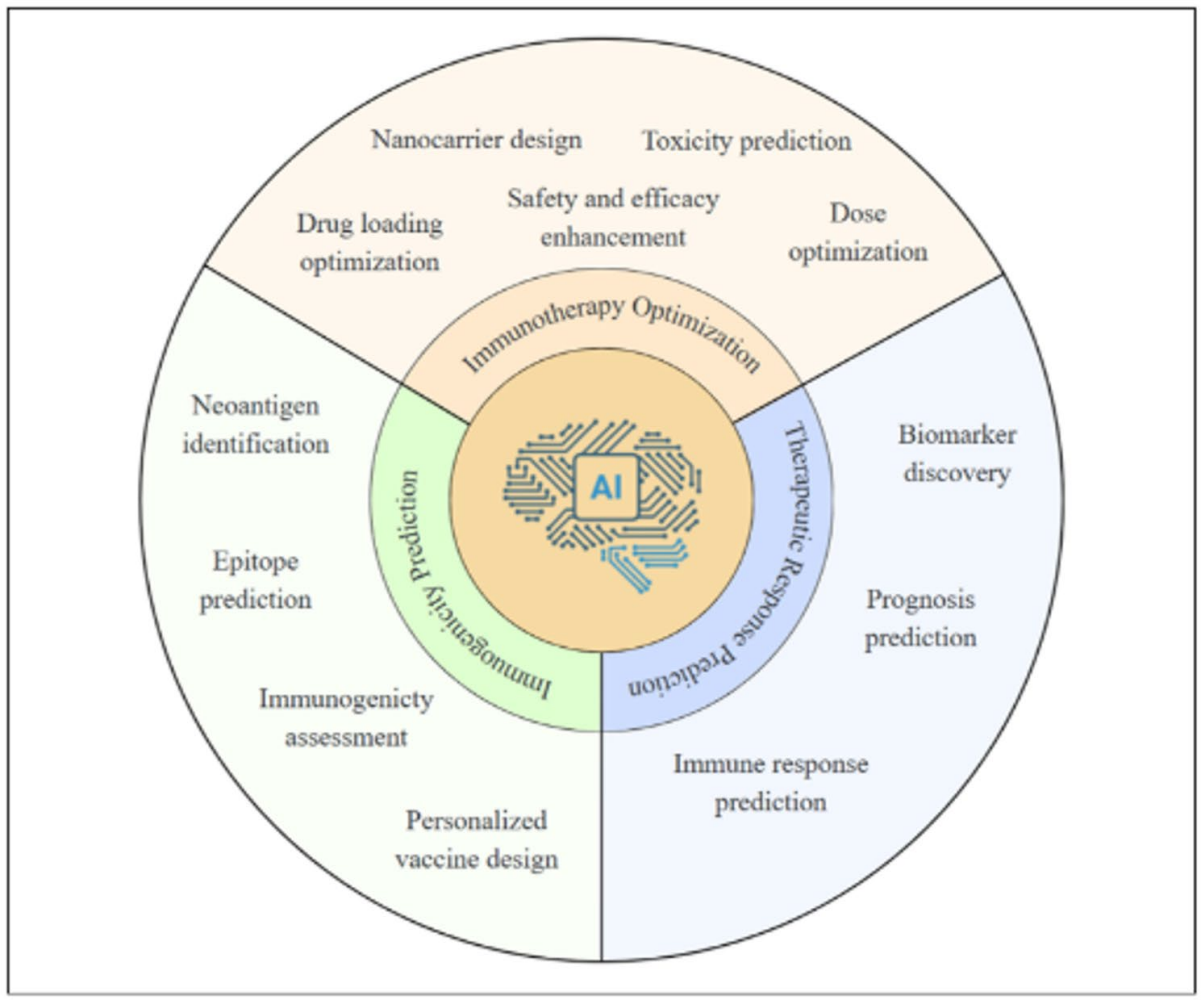
Schematic representation of AI-driven approaches in brain cancer nano-immunotherapy. AI algorithms can be utilized for antigen discovery and immunogenicity prediction, therapeutic response prediction, and optimization of NP formulations to improve treatment precision, efficacy and safety

AI, particularly through machine learning or deep learning, has greatly advanced NP-based immunotherapy, enhancing efficiency across optimization and clinical translation phases. In recent years, AI has shown potential to improve health care systems and has been applied across various medical fields. Within the field of immunotherapy, the application of AI is expanding rapidly, offering innovative and promising strategies for data-driven medical evaluation and treatment optimization [182]. By leveraging machine learning algorithms, large and complex datasets from various biological and clinical databases can be analyzed to improve the design and performance of immunotherapeutic approaches, antigen discovery, therapeutic response prediction, and nanocarrier optimization, as illustrated in Fig. 10 [183, 184]. Although a few studies have recently begun to explore AI applications in glioblastoma, research in this area remains limited. With continued advancements, this platform may be further developed and applied to glioblastoma to enhance therapeutic precision and improve overall cancer care outcomes.

### AI-assisted antigen discovery and immunogenicity prediction

In immunotherapy, immunogenicity is very important to ensure that an effective immune response is triggered against tumor cells. Integrating AI into immunotherapy development has significantly accelerated antigen discovery, enhanced predictive accuracy, and optimized experimental workflows. In a systematic review, Villanueva-Flores et al. found that AI has fundamentally reshaped immunogenicity prediction through epitope identification, moving from traditional, labor-intensive methods to highly accurate, efficient, and interpretable computational approaches [185]. Beyond epitope prediction, AI can also identify key determinants of tumor immunogenicity, a critical factor given the heterogeneity of tumors that often limits the success of immunotherapies. Charoentong et al. employed a random forest machine-learning approach to identify determinants of tumor immunogenicity and demonstrated superior predictive performance for identifying patients likely to respond to immunotherapies, particularly CTLA-4 and PD-1 blockade. They also proposed an immunophenoscore, measuring the overall immunogenicity of the tumor, which is important to predict the status of the immune response [186]. Notably, AI-based predictions of immunogenicity have shown accuracy similar to laboratory assays, indicating that deep learning models can help or even partly replace some experimental testing, thereby reducing the time and effort needed during early stages of immunotherapy development.

The ability of AI to identify highly immunogenic tumor antigens has important implications for the development of nanotechnology-based immunotherapies. In nanovaccine design, accurate antigen selection is critical because the therapeutic efficacy of NP delivery systems depends not only on successful delivery but also on the immunogenicity of the encapsulated antigen [187]. AI-driven neoantigen prediction can facilitate the identification of personalized glioma-associated antigens or epitopes for incorporation into nanocarrier and immunotherapy platforms [188, 189]. By prioritizing antigens with high immunogenic potential, AI may improve immunotherapy efficacy while reducing the experimental burden associated with antigen screening and validation. Consequently, the integration of AI-assisted antigen discovery with nanotechnology-based delivery systems offers a promising strategy for the development of personalized brain cancer immunotherapies.

### AI-based prediction of therapeutic response

Machine learning can extract meaningful patterns from immunological and clinical data through trained algorithms, making it a powerful tool for predicting therapeutic responses to immunotherapy [190, 191]. To evaluate immune responses, there are several approaches, including imaging, histopathology analysis, biomarker identification, genomics, multi-omics, transcriptomic, and proteomic data analysis [192–194]. By integrating these diverse data sources, AI models can identify complex relationships between tumor characteristics, immune status, and clinical outcomes that may not be readily detectable through conventional analytical methods.

AI algorithms also help in the immune response through quantification of the binding affinity and immunogenicity of T cell epitopes, which are antigenic fragments that can interact with antibodies or MHC molecules and elicit T cell responses [195]. For example, Hopp et al. discovered a machine learning method using self-organizing maps to identify gene regulatory modes governed by DNA methylation and expression in glioblastoma, providing valuable insights for predicting responses to ICIs [196]. Such findings highlight the ability of AI to analyze complex biological datasets and uncover predictive biomarkers that can support treatment selection and outcome prediction.

Furthermore, AI-guided prediction of therapeutic responses can facilitate personalized treatment strategies and improve clinical decision-making. Recent studies have shown that AI networks can predict immunotherapy outcomes based on genetic mutation profiles and other tumor-specific characteristics [197]. Similarly, Su et al. demonstrated the use of automated machine learning to investigate the feasibility of predicting H3 K27M gene mutation in patients with midline gliomas [198]. As H3 K27M status is closely associated with prognosis and overall survival, accurate prediction of this mutation may provide clinically relevant information for patient stratification and treatment planning [199].

The integration of AI-based therapeutic response prediction with nanotechnology-based immunotherapy offers additional opportunities for precision medicine. By combining molecular, imaging and immune-related data, AI models may help identify patients who are most likely to benefit from NP-mediated drug delivery systems, nanovaccines, and other immune-modulating nanocarriers [200, 201]. Such predictive capabilities could improve patient selection, optimize treatment strategies, and enhance the clinical efficacy of nanotechnology-based immunotherapies for brain cancer.

### AI-guided optimization of nanotechnology-based immunotherapy

The efficacy of immunotherapy modalities can be further enhanced through the integration of AI, which allows for the analysis of immunological biomarker data obtained after treatment. For example, in vaccine development, AI can assist in determining optimal dosing strategies based on efficacy and immune response data, thereby improving therapeutic outcomes while minimizing adverse effects [202]. Beyond dosage optimization, AI holds great potential to improve the overall design of immunotherapies by enhancing their immunogenicity, improving distribution and ensuring better safety profiles.

In nanotechnology-based immunotherapy, AI can facilitate the rational design and optimization of nanocarriers by predicting critical physicochemical properties that influence therapeutic performance [203–205]. Parameters such as particle size, surface charge, drug-loading efficiency, release kinetics, biodistribution, and BBB penetration play essential roles in determining the efficacy of NP-mediated immunotherapies [206, 207]. By analyzing large experimental and biological datasets, machine-learning algorithms can identify optimal NP characteristics and predict their behavior within complex biological environments, reducing the reliance on conventional trial-and-error approaches and accelerating formulation development [178].

Additionally, AI can be utilized to predict interactions between nanomaterials and biological systems, which is particularly important for assessing safety and biocompatibility [208]. Since certain nanomaterials may induce undesirable toxicities, AI-based models can help forecast potential biological responses and guide the selection of safer and more effective formulations [209]. For example, Lacinski et al. used machine learning methods to optimize nanostructured lipid carriers and identify the physicochemical parameters that affect the performance and safety of the formulation in glioblastoma. This study demonstrated that machine learning could uncover relationships between NP composition and cellular uptake that would be difficult to identify through conventional experimentation and successfully highlighted the elements that actively engage in the formation of effective drug carriers [210].

In the future, the integration of various AI technologies offers exciting opportunities to advance nano-immunotherapy for brain cancer. However, despite the remarkable promise of AI technologies, several critical challenges remain unresolved. Issues related to data privacy, patient consent, and ethical governance must be carefully addressed to ensure the responsible application of AI in healthcare settings [211]. Furthermore, because AI systems rely heavily on the availability and quality of existing datasets, the use of big data analytics introduces additional complexities. The integration of large and heterogeneous datasets from diverse sources can compromise data quality and consistency, ultimately affecting predictive accuracy and model reliability [212]. Overcoming these challenges is essential to fully leverage the potential of AI-driven nano-immunotherapy in improving brain cancer care.

## Conclusion

Over the years, deeper insights into the mechanisms by which cancer cells escape immune surveillance have led to substantial advancements in cancer immunotherapy. However, in brain tumors, factors such as tumor heterogeneity, the immunosuppressive TME, and the restrictive nature of the BBB continue to limit treatment effectiveness. Despite the challenges, immunotherapy still holds promise for the treatment of brain tumors. Combinatorial strategies that integrate immunotherapy with conventional modalities such as radiotherapy and chemotherapy are being actively explored to achieve synergistic effects and improve clinical outcomes.

In the coming years, immunotherapy research is rapidly evolving toward precision and personalization, aiming to enhance specificity, safety, and therapeutic efficacy. To further optimize immune-based treatments, innovative delivery platforms, particularly nanotechnology-based, are also being developed to enhance stability, bioavailability, and targeted delivery across the BBB. Among these strategies, nanotechnology-assisted cancer vaccines have emerged as a highly promising approach, offering the potential to induce durable immune memory, enhance antigen presentation, and achieve long-term tumor control.

Ongoing preclinical and clinical research highlight the potential of NPs as advanced delivery platforms for a wide range of therapeutic applications in oncology, neurology, infectious diseases, and regenerative medicine. These advances demonstrate exceptional promise for brain cancer therapy, owing to their ability to encapsulate drugs efficiently, enable targeted delivery, penetrate the BBB, and facilitate combination treatments. Thus, the integration of nanotechnology with cancer vaccines and other immunotherapies represents a promising frontier in achieving durable and precise treatment for brain tumors in the future.

Nanotechnology itself provides advanced delivery platforms that enhance the stability, bioavailability, and immunogenicity of the immunotherapeutic agents through precise delivery, controlled release, and targeted interaction with immune cells. When combined with AI, the overall efficacy of NP-based immunotherapy can be further improved, as AI assists in optimizing therapy design, predicting and monitoring immune responses, and reducing experimental uncertainty.

Despite ongoing challenges concerning data privacy, ethical governance, and model transparency, the rapid progress of AI-driven platforms underscores their immense potential to transform brain cancer care. With ongoing advancements, these technologies are expected to further enhance their contribution to the design and development of future immunotherapeutic strategies, offering treatments that are not only more effective and safer but also more accessible to patients worldwide.

## Supplementary Information

Supplementary Material 1.

## Data Availability

All data supporting this study are contained within the article.
